# IL-24 intrinsically regulates Th17 cell pathogenicity in mice

**DOI:** 10.1084/jem.20212443

**Published:** 2022-07-12

**Authors:** Christopher Sie, Ravi Kant, Christian Peter, Andreas Muschaweckh, Monika Pfaller, Lucy Nirschl, Helena Domínguez Moreno, Tereza Chadimová, Gildas Lepennetier, Tanja Kuhlmann, Rupert Öllinger, Thomas Engleitner, Roland Rad, Thomas Korn

**Affiliations:** 1 Institute for Experimental Neuroimmunology, Technical University of Munich School of Medicine, Munich, Germany; 2 Institute of Neuropathology, University Hospital Münster, Münster, Germany; 3 Institute of Molecular Oncology and Functional Genomics, TranslaTUM Cancer Center, Technical University of Munich School of Medicine, Munich, Germany; 4 Department of Neurology, Technical University of Munich School of Medicine, Munich, Germany; 5 Munich Cluster for Systems Neurology (SyNergy), Munich, Germany

## Abstract

In certain instances, Th17 responses are associated with severe immunopathology. T cell–intrinsic mechanisms that restrict pathogenic effector functions have been described for type 1 and 2 responses but are less well studied for Th17 cells. Here, we report a cell-intrinsic feedback mechanism that controls the pathogenicity of Th17 cells. Th17 cells produce IL-24, which prompts them to secrete IL-10. The IL-10–inducing function of IL-24 is independent of the cell surface receptor of IL-24 on Th17 cells. Rather, IL-24 is recruited to the inner mitochondrial membrane, where it interacts with the NADH dehydrogenase (ubiquinone) 1 α subcomplex subunit 13 (also known as Grim19), a constituent of complex I of the respiratory chain. Together, Grim19 and IL-24 promote the accumulation of STAT3 in the mitochondrial compartment. We propose that IL-24–guided mitochondrial STAT3 constitutes a rheostat to blunt extensive STAT3 deflections in the nucleus, which might then contribute to a robust IL-10 response in Th17 cells and a restriction of immunopathology in experimental autoimmune encephalomyelitis.

## Teaser

Th17 cells restrain their own pathogenicity by producing IL-24, which induces IL-10 in a cell-intrinsic manner. IL-24 creates a STAT3 sink at the inner mitochondrial membrane and contributes in redirecting STAT3 from the nucleus to the mitochondrial compartment.

## Graphical Abstract

**Figure:**
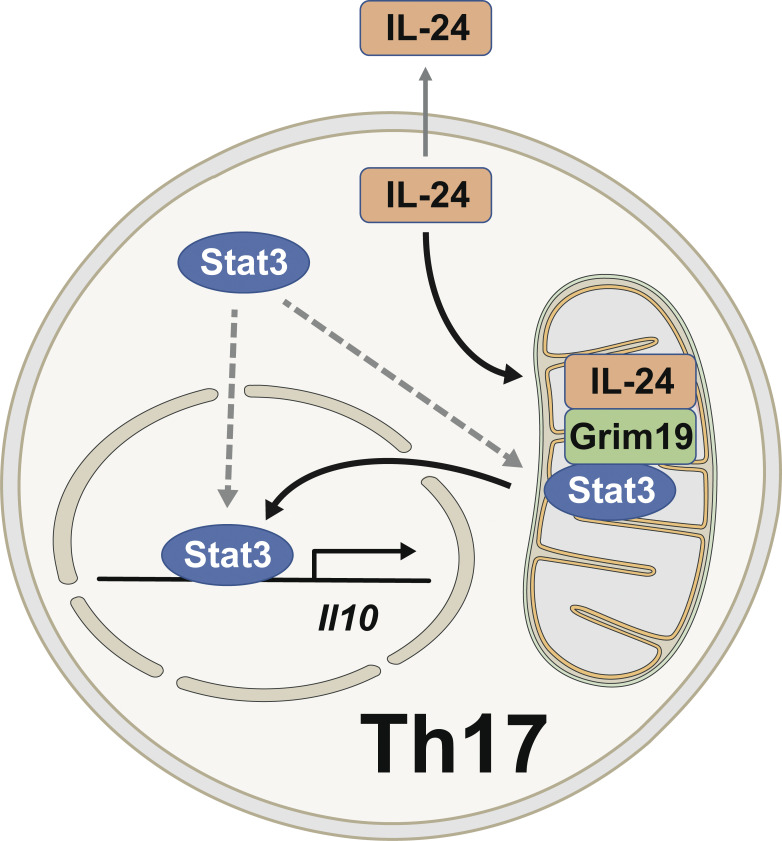

## Introduction

T helper 17 (Th17) cells have been established as a distinct lineage of T helper cells, which orchestrate host defense at epithelial barriers, in particular against fungi and distinct extracellular bacteria (Korn et al., 2009). Because of the broad tissue response to cytokines produced by Th17 cells, the extent of immunopathology in Th17-mediated immune reactions can be dramatic, and they have been involved in the pathogenic cascade in a variety of autoimmune diseases including psoriasis (Lowes et al., 2008), rheumatoid arthritis (Sato et al., 2006), and multiple sclerosis (Brucklacher-Waldert et al., 2009). Adaptive immunopathology is tightly regulated, and effector T cell intrinsic expression of IL-10 has long been identified as an important means to autoregulate effector T cell responses (Mathisen et al., 1997). For instance, in *Toxoplasma gondii* infection and tuberculosis, expression of IL-10 by Th1 cells in a STAT4-dependent manner in response to sustained exposure to IL-12 is critical to restrict exaggerated immunopathology (Jankovic et al., 2010). Effector T cell intrinsic regulation has also been described for Th17 cells, which respond to IL-12 and IL-27 by upregulating IL-10 in a Blimp1-dependent manner (Heinemann et al., 2014). However, neither IL-12 nor IL-27 is produced by T cells themselves; they need to be provided by the inflammatory environment (primarily by myeloid cells) to induce downmodulatory pathways in T cells. In contrast, imprinting of IL-10 production in Th2 cells requires repetitive stimulation in the presence of IL-4 (an intrinsic product of Th2 cells) and is thus independent of exogenous factors, constituting a T cell–autonomous regulatory loop (Lohning et al., 2003).

Here, we wondered whether similar pathways of effector T cell–intrinsic regulation of IL-10 also exist in the context of sustained Th17 cell responses. IL-24 has been identified in the transcriptional module of nonpathogenic Th17 cells (Gaublomme et al., 2015). IL-24 belongs to the IL-20 family of cytokines and is produced by T cells and monocytes, but also by nonhematopoietic cells such as melanocytes and endothelial cells (Rutz et al., 2014). IL-24 signals through a receptor complex composed of IL-20Rα and IL-20Rβ (Logsdon et al., 2012). The same receptor complex is also used by the close IL-24 relatives IL-19 and IL-20. Alternatively, IL-24 (like IL-20 but not IL-19) can signal through the IL-22Rα1/IL-20Rβ receptor complex (Logsdon et al., 2012). Genetic ablation of *Il20rb* indicated that IL-20Rβ signaling in T cells decreases the production of IL-2 and IFN-γ and increases IL-10 upon antigen-specific stimulation, identifying IL-24 as a rather downregulatory cytokine (Wahl et al., 2009). In addition, IL-24 lowered GM-CSF production in T cells in a SOCS3-dependent manner (Chong et al., 2020). Although *Il20rb*^−/−^ mice did not show a phenotype in *Citrobacter rodentium* infection (Zheng et al., 2008), another strain of *Il20rb*^−/−^ mice showed aggravated contact hypersensitivity reactions, supporting an immunomodulatory function of IL-20 family cytokines (Wahl et al., 2009). IL-20Rβ is also expressed in nonhematopoietic cells. For instance, in keratinocytes and astrocytes, IL-24 inhibits the production of IL-1β and IL-6, respectively (Burmeister et al., 2019; Myles et al., 2013). Therefore, IL-24 can drive an immunoregulatory feedback loop independent of adaptive immune cells. Notably, IL-19 and IL-20 but not IL-24 induce hyperkeratosis in response to IL-23 (Chan et al., 2006), suggesting that IL-24 does not appear to be associated with dysfunctional tissue responses. IL-24 is proapoptotic in tumor cells through downregulation of Bcl-xL and induction of p53 (Yacoub et al., 2004). Interestingly, a nonsecretable form of IL-24 had effects similar to those of the secreted full-length isoform of IL-24 (Sauane et al., 2004), and therefore, an intracellular function of IL-24 independent of its membrane receptor has been proposed. In yeast two-hybrid-screens, IL-24 physically interacted with Grim19 (also called Ndufa13), a subunit of the NADH dehydrogenase 1 (complex I) of the electron transport chain located at the inner mitochondrial membrane (Hu et al., 2016). Grim19, in turn, was shown to also interact with STAT3 and target it into the inner mitochondrial membrane, where it modulates cellular respiration (Gough et al., 2009; Tammineni et al., 2013).

In this study, we reveal that IL-24 exerts a fundamental function in T cells independent of its surface receptor IL-20Rβ. By interacting with Grim19 and promoting the recruitment of STAT3 to mitochondria, intracellular IL-24 contributes to channeling STAT3 away from its regular nuclear signal transduction pathway. Therefore, as an integral part of the Th17 portfolio of cytokines, IL-24 establishes a Th17 cell–intrinsic negative regulatory loop. Targeting IL-24 in gain- and loss-of-function approaches may provide a therapeutic rheostat of ongoing Th17 responses.

## Results

### IL-24 is expressed in nonpathogenic and pathogenic Th17 cells

**Figure 1.**
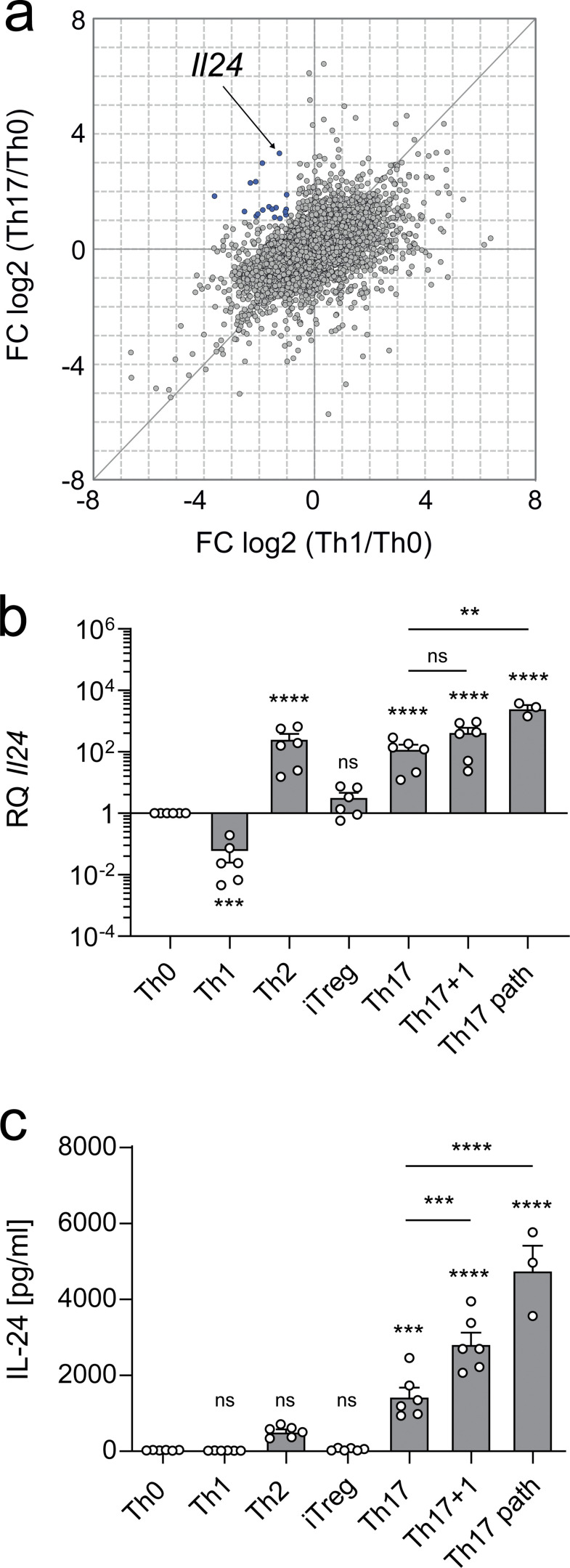
**IL-24 segregates with the Th17-associated portfolio of cytokines. (a)** Naive T cells were differentiated into Th0, Th1, or Th17 cells (TGF-β plus IL-6). Their transcriptome was assessed by microarray analysis (Heinemann et al., 2014). **(b and c)** The indicated T cell subsets were differentiated for 3 d from naive T cells in vitro, followed by analysis of RNA and culture supernatants by RT-qPCR and ELISA, respectively. Both readouts show summarized data (mean ± SEM) from two independent experiments. Asterisks indicate significance level after one-way ANOVA followed by Sidak’s multiple comparison test (multiplicity-adjusted P values: ****, P < 0.0001; ***, P < 0.001; **, P < 0.01), with asterisks on top relating to results from tests against Th0 and black lines indicating individual results from tests against Th17. **(b)** Results from RT-qPCR using TaqMan assays, showing *Il24* normalized to *Actb* expression with log-transformed values relative to Th0. **(c)** Secretion of IL-24 into the supernatant as assessed by ELISA.

Gene expression of *Il24* has been reported to be increased in Th17 cells differentiated with TGF-β plus IL-6 compared with Th0 culture conditions (Yosef et al., 2013). While it has been argued that expression of *Il24* is a feature of nonpathogenic Th17 cells (Gaublomme et al., 2015) as compared to pathogenic Th17 cells (Ghoreschi et al., 2010), the function of IL-24 in T cells has not been systematically investigated. When revisiting our own data from a previous study (Heinemann et al., 2014), we confirmed that IL-24 was expressed in Th17 cells differentiated from naive T cells with TGF-β plus IL-6, but not in Th1 cells (Fig. 1 a). To assess the expression of IL-24 in different Th cell subsets, we sorted naive T cells from murine spleen and differentiated them to Th1, Th2, induced T regulatory (iTreg), and Th17 cells using the indicated cytokine cocktails. Th17 cells were differentiated either with TGF-β plus IL-6 (nonpathogenic Th17 cells) in the presence or absence of IL-1β or with IL-6 plus IL-23 plus IL-1β (in the absence of TGF-β, pathogenic Th17 [Th17path] cells). Although Th1 cells did not express IL-24, *Il24* mRNA and secreted protein were detected in Th2 and Th17 cells (Fig. 1, b and c). Th17path cells contained even higher amounts of IL-24 mRNA and secreted more IL-24 into the supernatant than nonpathogenic Th17 cells (Fig. 1, b and c), suggesting that IL-24 expression was not a discriminating feature between these Th17 cell subsets.

### The expression of IL-24 in T cells is regulated in a transcriptional and posttranscriptional manner

**Figure 2.**
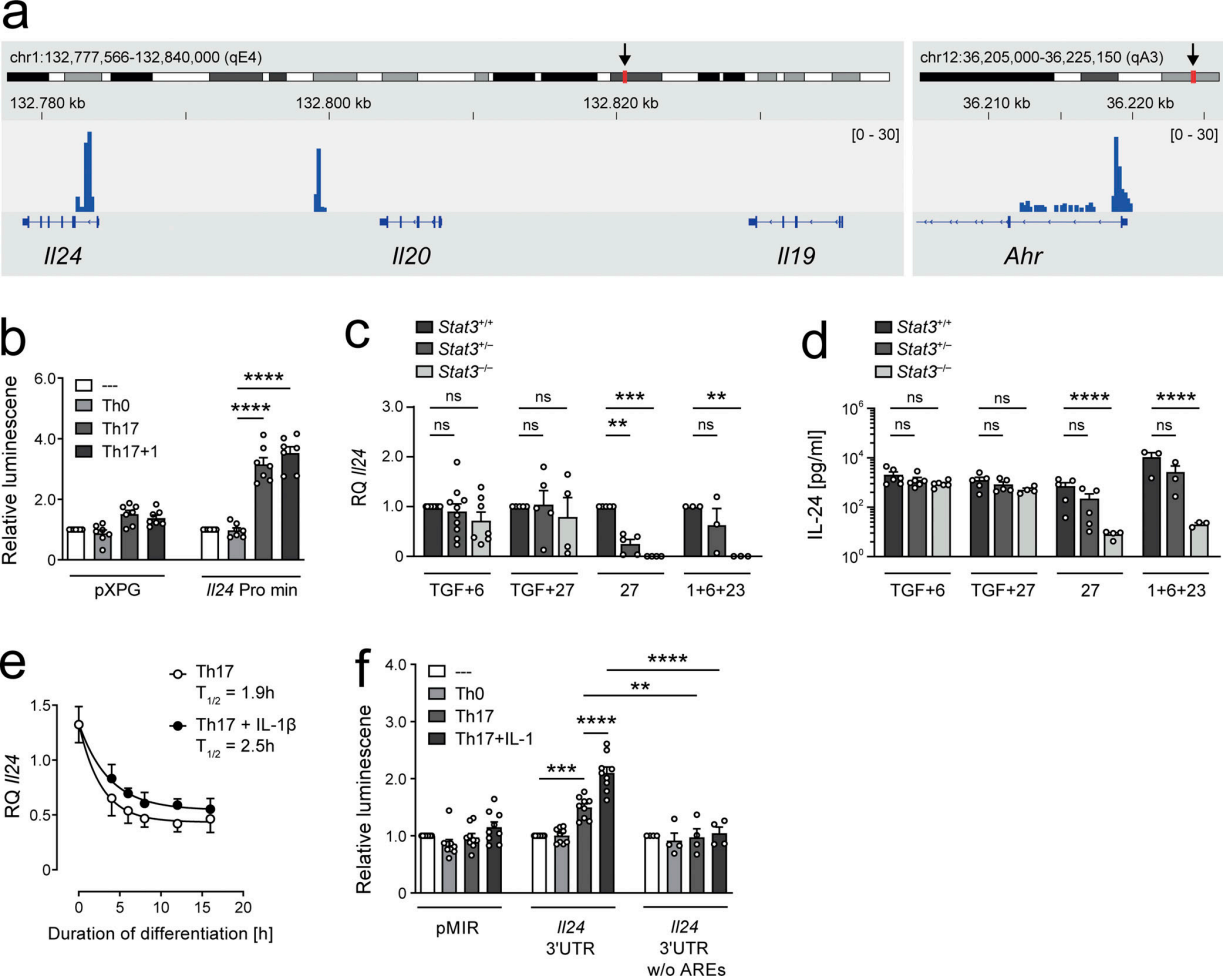
**IL-24 expression in T cells is regulated in a transcriptional and posttranscriptional manner. (a)** ChIP-seq track (GSM540722, mm9) of the *Il24* locus (left) after precipitation with anti-pSTAT3 in Th17 cells (data from Durant et al., 2010) with the *Ahr* promoter (right) shown on the same scale for reference. **(b)** *Il24* minimal promoter assay summarizing normalized data from seven independent experiments (mean ± SEM). EL4 murine thymoma cells were transfected with pXPG vector with or without the *Il24* minimal promoter and costimulated with plate-bound anti-CD3 and soluble anti-CD28 for 48 h in the presence of cytokines to induce the indicated Th subsets. Cells were stimulated for 4 h with PMA/ionomycin and then lysed. Relative luminescence of luciferase activity normalized to renilla. Asterisks indicate significance level for selected tests against the Th0 subset as determined by Tukey’s multiple comparison test after one-way ANOVA (****, P < 0.0001). **(c and d)** In the presence of TGF-β, the expression of *Il24* is independent of STAT3. Naive T cells from *Stat3*^+/+^ wild-type C57BL/6, *Stat3*^+/−^, and *Stat3*^−/−^mice were sorted and costimulated for 3 d under standard Th17 conditions (TGF-β plus IL-6), Th17path conditions (IL-1, IL-6, and IL-23), or Tr-1 conditions (TGF-β plus IL-27 or IL-27 alone). **(c)** RT-qPCR quantification of *Il24* expression normalized to *Actb* and relative to the wild-type expression level for each condition. Summary of normalized data from five independent experiments (mean ± SEM). **(d)** Secretion of IL-24 into the supernatant as assessed by ELISA, summary of three independent experiments (mean ± SEM; log-transformed data). Asterisks indicate significance level of Dunnett’s multiple comparison test after two-way ANOVA (****, P < 0.0001; ***, P < 0.001; **, P < 0.01). **(e)** IL-1β stabilizes *Il24* mRNA. Decay of *Il24* mRNA under actinomycin D treatment as measured by RT-qPCR in EL4 cells differentiated toward Th17 cells for 72 h and then followed for 16 h in the presence or absence of IL-1β. Half-life was calculated using nonlinear regression (one-phase decay). Data is representative of two independent experiments, with mean ± SEM of three biological replicates per group depicted. **(f)** Luciferase reporter activity in EL4 cells under various Th conditions after 48 h of culture, transfected with empty pMIR-GLO or vector containing *Il24* 3′ UTR with and without AREs in the reporter construct. Relative luminescence of luciferase activity normalized to renilla. Summary of normalized data from four independent experiments (mean ± SEM). Asterisks indicate significance level as determined by Tukey’s multiple comparison test after one-way ANOVA (****, P < 0.0001; ***, P < 0.001; **, P < 0.01).

The induction of IL-24 in both nonpathogenic and pathogenic Th17 cells suggested that the STAT3 pathway might be involved in the induction of IL-24, since STAT3 activation is common in the developmental program of these T cell subsets (Hirahara et al., 2015) and is also required for the generation of Th2 cells (Stritesky et al., 2011), which, in contrast to Th1 cells, express high amounts of IL-24. STAT3 chromatin immunoprecipitation sequencing (ChIP-seq) indicated that STAT3 bound to *Il24* to a similar extent as to a classic STAT3 target such as *Ahr* (Fig. 2 a), and a luciferase construct bearing the minimal promoter of *Il24* transfected in EL4 cells responded to the stimulation with TGF-β plus IL-6, which induces STAT3, but not to stimulation conditions in Th0 cultures (Fig. 2 b). However, when we tested naive *Stat3*^+/−^ T cells or *Stat3*^−/−^ T cells, differentiated under Th17 conditions (TGF-β plus IL-6), we did not observe differences in *Il24* mRNA or secreted IL-24 protein compared with wild-type T cells as long as TGF-β was present (Fig. 2, c and d), whereas STAT3 was absolutely necessary for the induction of *Il24* in the absence of TGF-β, such as upon stimulation with IL-27 (Tr-1 condition) or in Th17path conditions (Fig. 2, c and d).

Th17path cells were more abundant in *Il24* mRNA than nonpathogenic Th17 cells, yet *Il24* promoter activity was not further enhanced by IL-1β compared with stimulation with TGF-β plus IL-6 (Fig. 2 b). Therefore, we considered whether IL-1β could exert a posttranscriptional regulation of *Il24* mRNA. IL-1β can control the stability of mRNAs by inducing and activating mRNA binding proteins (Cok et al., 2003). To test this possibility, we measured the kinetics of *Il24* mRNA decay in EL4 cells under conditions of blockade of de novo gene transcription with actinomycin D. In the presence of IL-1β, the decay of *Il24* mRNA was delayed, suggesting that IL-1β enhanced the stability of *Il24* mRNA in EL4 cells cultured with TGF-β plus IL-6 (Fig. 2 e). It is a common feature of RNA-stabilizing proteins to bind to AU-rich elements (AREs) in the 3′ UTR of RNAs (Keene, 2007). Therefore, we nucleofected EL4 cells with a luciferase construct comprising either the intact 3′ UTR of *Il24* or a 3′ UTR lacking AREs. When AREs were eliminated from the *Il24* construct, the *Il24* RNA-increasing effect of IL-1β was abolished (Fig. 2 f). Taken together, these data indicated that IL-1β increased the abundance of *Il24* mRNA by increasing its stability.

### IL-24 is expressed in Th17 cells in vivo

**Figure 3.**
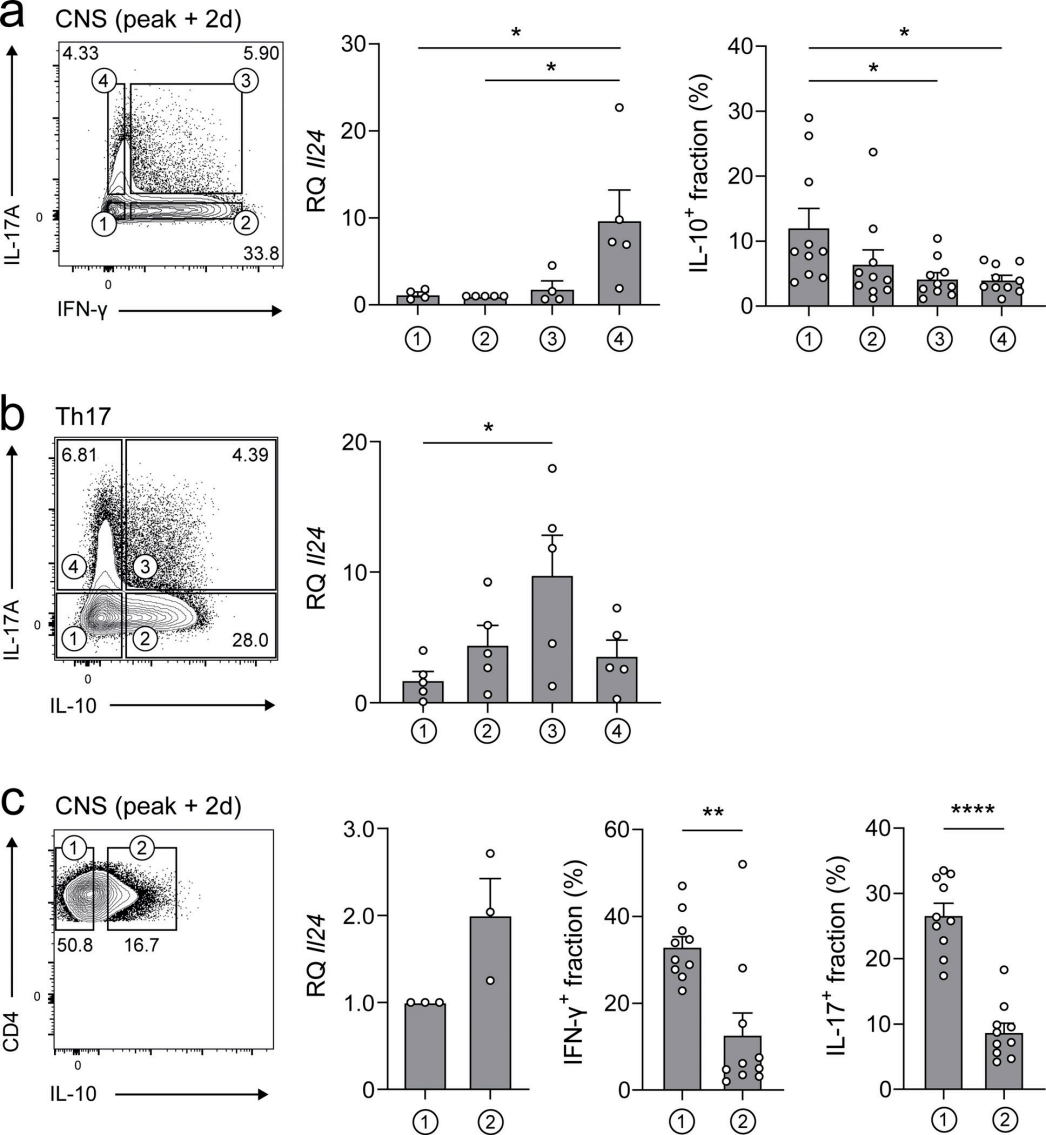
**IL-24 is coexpressed with IL-17 and IL-10. (a)** *Il24* expression segregates with IL-17 in vivo. Smart × Great reporter mice, in which IL-17A is reported by hNGFR and IFN-γ by YFP, were immunized for EAE, and 2 d after the peak of disease, CD4^+^ T cells were isolated from the inflamed CNS and sorted for the indicated cytokine-producing subpopulations, and each of them was subjected to RT-qPCR analysis for relative expression of *Il24*, normalized to *Actb*. Summarized data from two independent experiments (mean ± SEM). For quantification of IL-10–producing fractions among the indicated IL-17A/IFN-γ populations, Smart × Great mice were crossed with IL-10 (GFP) reporter mice and subjected to EAE, followed by flow cytometric analysis of CNS infiltrates (10 biological replicates, mean ± SEM). Asterisks indicate significance level of Tukey’s multiple comparison test (*, P < 0.05). **(b and c)** IL-24 is coexpressed with IL-17 and IL-10 in vitro and in vivo. **(b)** In vitro differentiation of naive CD4^+^ T cells from IL-17A (hNGFR) × IL-10 (GFP) reporter mice into Th17 cells for 3 d, sorted for the indicated cytokine-producing subpopulations, and subjected to RT-qPCR analysis of *Il24*, normalized to *Actb*. Summary of two independent experiments (mean ± SEM), with asterisks indicating significance level of Tukey’s multiple comparison test (*, P < 0.05). **(c)** T cells from IL-10 (GFP) reporter EAE mice, 2 d after peak, were sorted according to IL-10 signal and subjected to RT-qPCR analysis of *Il24* normalized to *Actb*. Summarized data from two independent experiments (mean ± SEM). For quantification of IFN-γ– and IL-17A–producing cells in the indicated IL-10 populations, IL-10 reporter mice were crossed to Smart × Great reporter mice and subjected to EAE and cytometry as outlined above (10 biological replicates, mean ± SEM). Asterisks indicate significance level of two-tailed *t* tests (****, P < 0.0001; ** P < 0.01).

**Figure S1.**
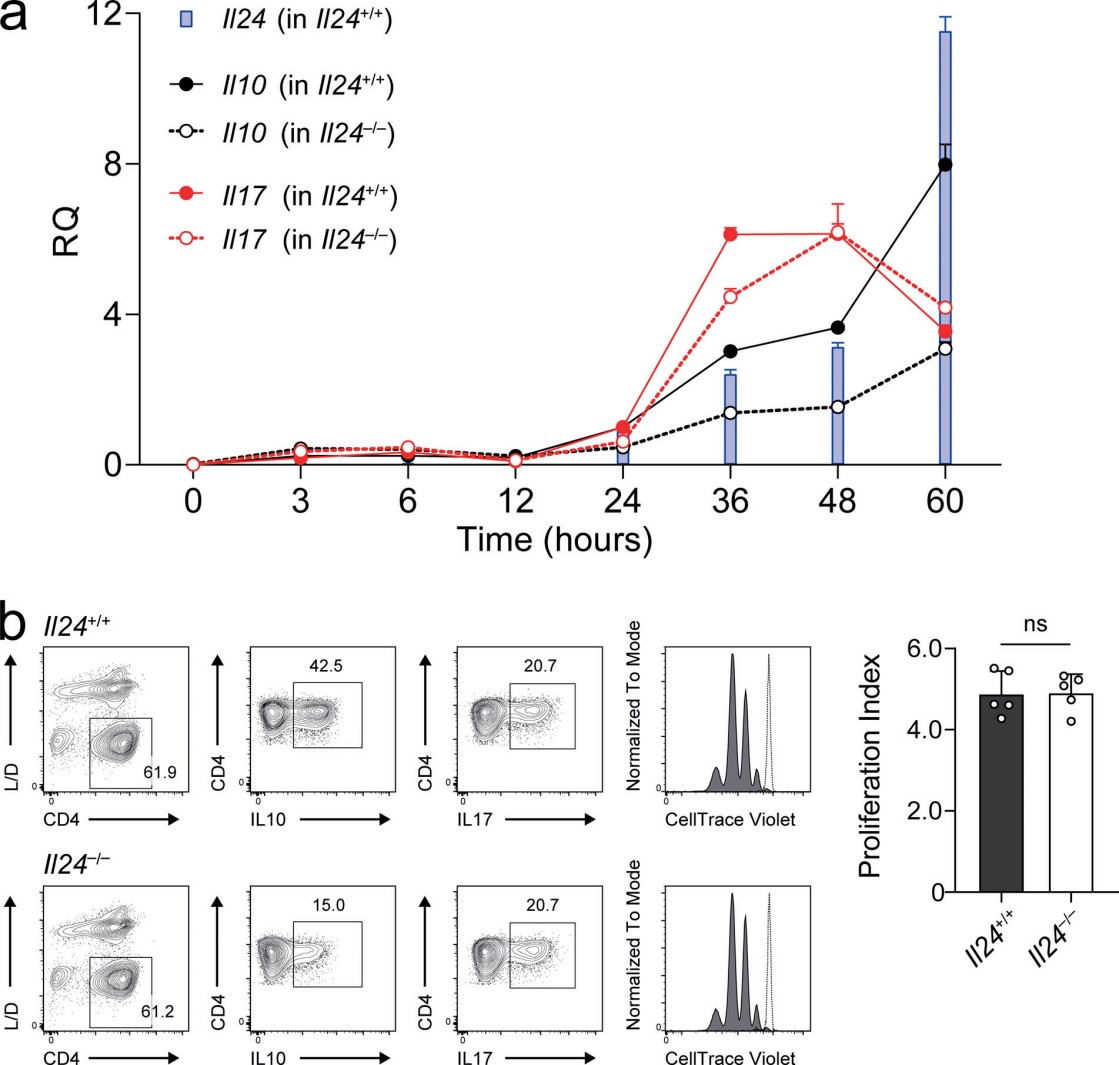
**IL-24 is a late cytokine and has no effect on the proliferation of Th17 cells. (a)** Kinetics of cytokine expression in Th17 cells. Naive *Il24*^+/+^ wild-type (solid lines; filled bars and circles) and *Il24*^−/−^ (dashed lines; empty circles) T cells were isolated and cultured in Th17 conditions for the indicated periods with three technical replicates per time point (mean ± SD), followed by RT-qPCR for *Il10* (black), *Il17* (red), and *Il24* (blue). **(b)** Naive IL-24–sufficient or –deficient T cells were differentiated into Th17 cells and tested for cytokine production and proliferation by dye dilution on day 3 of the differentiation culture. Representative flow cytometry plots (left) with CellTrace Dye of labeled, proliferated cells (filled histograms) compared with labeled, unstimulated cells after the same time of culture (dotted lines). Absence of statistical significance as indicated was tested using a two-tailed *t* test on the proliferation index (right) summarized from two independent experiments (mean ± SEM).

Next, we wanted to test whether Th17 cells expressed IL-24 in vivo. Therefore, experimental autoimmune encephalomyelitis (EAE) was induced by immunization with myelin oligodendrocyte glycoprotein (MOG(35–55)) in a reporter mouse strain, in which IFN-γ–producing T cells are labeled by YFP (Reinhardt et al., 2009) and IL-17–producing T cells by hNGFR (Price et al., 2012). CD4^+^ T cells were isolated from the central nervous system (CNS) and sorted directly ex vivo according to their expression of IFN-γ and IL-17. *Il24* mRNA was most abundant in IL-17^+^ T cells but absent in IFN-γ^+^ single-positive T cells (Fig. 3 a). IL-24 is a “late” cytokine in Th17 cells (Fig. S1 a). Therefore, we sorted T cells from the CNS 2 d after the peak of EAE. At that time point, the fraction of IL-17A/IFN-γ double producers was already very low, potentially affecting the robustness of the *Il24* measurement in the IL-17A/IFN-γ double-producing compartment (Fig. 3 a). Notably, *Il24* and *Il10* were coexpressed in IL-17A single producers but not the IFN-γ single producers (Fig. 3 a). Because IL-24 production segregated with IL-10 production by Th17 cells in vitro (Fig. 3 b), we used IL-10 (GFP) reporter mice (Madan et al., 2009) to isolate IL-10 GFP^+^ T cells from the CNS shortly after the peak of EAE and tested their potential to produce IL-24. *Il24* mRNA was more prominently expressed in IL-10 GFP^+^ than in IL-10 GFP^−^ T cells (Fig. 3 c). Interestingly, even though fewer than in the IL-10 GFP^−^ T cell fraction, IL-10 GFP^+^ T cells comprised both IFN-γ producers and IL-17 producers (Fig. 3 c, right panels). These data suggested that IL-24 was expressed in Th17 cells in vivo and was associated with effector T cells that acquired the capacity to produce IL-10. Because Th1 cells do not express IL-24, but are present among IL-10 producers, alternative IL-10–supporting pathways independent of IL-24 are likely operational in Th1 cells.

### IL-24 regulates IL-10 production in a T cell–intrinsic manner

**Figure 4.**
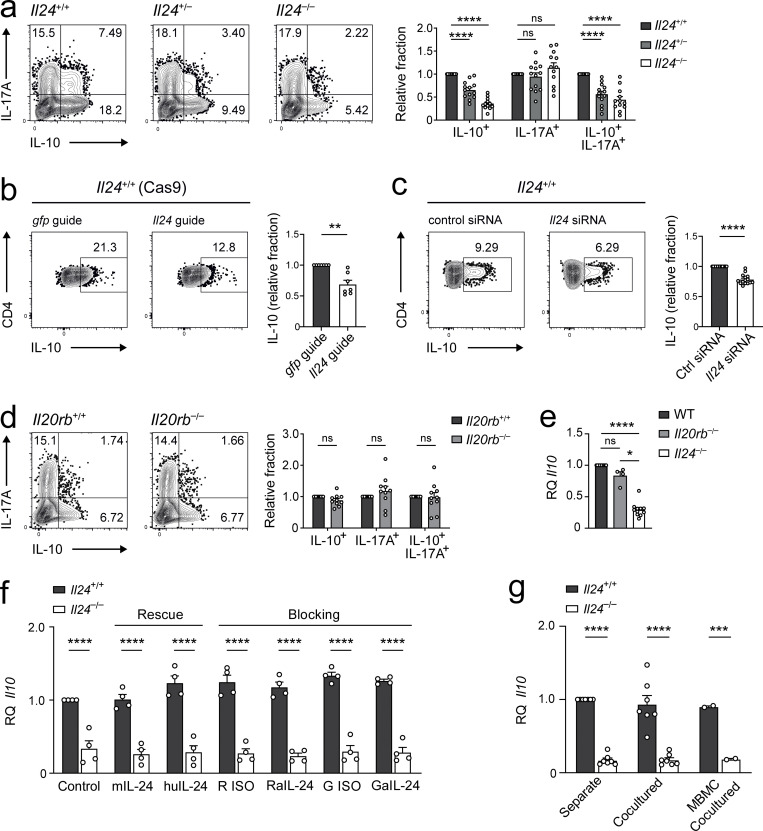
**The reduced IL-10 production in IL-24–deficient Th17 cells is a cell-intrinsic property. (a)** Gene dose effect through titrating down the genetic availability of *Il24* product directly impacts IL-10. Naive T cells from *Il24*^+/+^, *Il24*^+/−^, and *Il24*^−/−^ mice were cultured for 3 d under Th17 conditions and then subjected to intracellular cytokine staining for IL-17 and IL-10. Representative flow cytometry plots (left) and quantification of relative fractions of cells that were either single- or double-positive for the indicated cytokines, normalized to *Il24*^+/+^ levels for each population (right), summarizing 10 independent experiments (mean ± SEM). Relative fractions were derived by dividing the frequency of the indicated cytokine-expressing subset (among CD4^+^ cells) by the corresponding frequency of the *Il24*^+/+^ (wild-type) genotype for each experiment. Therefore, for each subset, the relative fraction for *Il24*^+/+^ itself was defined as 1, while a relative fraction lower than 1 indicates a decrease of that particular subset compared with the wild-type population in a given genotype and vice versa. Asterisks indicate significance level of Dunnett’s multiple comparison test following two-way ANOVA (****, P < 0.0001). **(b and c)** Acute ablation of IL-24 reduces IL-10 expression in Th17 cells. **(b)** Naive Cas9 transgenic T cells were cultured in Th17 differentiation conditions. After retroviral transduction with gRNAs targeting *Il24* or control (directed at the GFP transgene) on day 1, Th17 cells were analyzed for intracellular IL-10 on day 3. Representative FACS plots (left, pregated for viable and Thy1.1^+^ transduced CD4^+^ cells) and summary of four independent experiments (mean ± SEM) normalized to control (right). **(c)** Naive T cells from wild-type mice were cultured in Th17 differentiation conditions. Th17 cells were treated with siRNA targeting IL-24 or control siRNA (Ctrl) on day 1 and analyzed for intracellular IL-10 on day 3. Representative FACS plots (left, pregated for viable CD4^+^ cells) and summary (mean ± SEM) of four independent experiments (right). **(b and c)** Relative fraction indicates IL-10^+^ frequency (among CD4^+^ cells) for the respective treatment group divided by the corresponding IL-10^+^ frequency of the control group (*gfp* guide and control siRNA, respectively). Asterisks indicate significance level of two-tailed *t* tests (****, P < 0.0001; **, P < 0.01). **(d and e)** Absence of IL-24 receptors does not impact IL-10. Naive T cells from wild-type, *Il20rb*^−/−^, and *Il24*^−/−^ mice were cultured for 3 d under Th17 conditions and then subjected to (d) intracellular cytokine staining for IL-17 and IL-10 with representative cytometry plots (left, pregated for viable CD4^+^ T cells) and summary (mean ± SEM) of five independent experiments (right, indicated as relative fractions for each subset normalized to wild-type, as described above), as well as (e) RT-qPCR analysis for *Il10* expression, summarized from seven independent experiments with data normalized to wild-type expression levels (mean ± SEM). Asterisks indicate significance level (****, P < 0.0001; *, P < 0.05) of Sidak’s multiple comparison test (d) and Tukey’s multiple comparison test (e). **(f)** *Il10* expression is impervious to external IL-24. Naive T cells from *Il24*^+/+^ and *Il24*^−/−^ mice were cultured for 3 d under Th17 conditions in the presence or absence of exogenous murine or human recombinant IL-24, or anti–IL-24 antibodies from rat or goat or their respective isotype controls, followed by RT-qPCR for *Il10*. Summary of two independent experiments (mean ± SEM). **(g)** Availability of natively secreted IL-24 does not impact *Il10* expression in coculture. Naive T cells from congenically marked *Il24*^+/+^ and *Il24*^−/−^ as well as from mixed bone marrow chimeric mice with 1:1 ratio of the two compartments were cocultured for 3 d under Th17 conditions and then sorted apart for individual RT-qPCR analysis of *Il10* expression. Summary of seven independent experiments (mean ± SEM). In panels f and g, asterisks indicate significance levels of Sidak’s multiple comparison test (****, P < 0.0001; ***, P < 0.001).

To elucidate whether IL-10 production was mechanistically linked with the capacity of T cells to produce IL-24, we assessed the cytokine profile of Th17 cells differentiated from wild-type or IL-24–deficient naive T cells in the presence of TGF-β plus IL-6. While the fraction of IL-17 producers was identical in wild-type and *Il24*^−/−^ Th17 cells, IL-10^+^ cells were significantly reduced in *Il24*^−/−^ Th17 cells compared with their wild-type counterparts. We noticed a gene dose effect, since Th17 cells derived from naive heterozygous *Il24*^+/−^ T cells produced half as much IL-10 as wild-type Th17 cells, while IL-24–deficient Th17 cells exhibited largely reduced IL-10 production (Fig. 4 a). The proliferation of IL-24–sufficient and –deficient Th17 cells was similar despite their differential production of IL-10 (Fig. S1 b). However, both IL-24 and IL-10 were found to be late cytokines during T cell activation compared with IL-17 (Fig. S1 a), and the increase in IL-24 production in Th17 cells coincided with the growing difference in IL-10 production between wild-type and IL-24–deficient Th17 cells.

We used sorted naive (CD4^+^CD44^low^CD62L^high^CD25^−^) T cells for our differentiation cultures. Yet it was possible that wild-type and *Il24*^−/−^ naive T cells had different transcriptional pre-imprintings. Therefore, we sought to investigate whether acute loss of IL-24 in mature T cells would also modulate their capacity to produce IL-10 during Th17 differentiation. Indeed, disruption of the *Il24* locus by CRISPR/Cas9 in wild-type (Cas9 transgenic) T cells diminished their production of IL-10 upon Th17 differentiation with TGF-β plus IL-6 compared with Cas9 transgenic T cells transduced with an irrelevant control single-guide RNA (gRNA; Fig. 4 b). In contrast, the capacity of CRISPR/Cas9-engineered IL-24–deficient T cells to produce IL-17 or GM-CSF was not increased compared with control T cells (not depicted). The gene loci for *Il24* and *Il10* are both on chromosome 1 at a distance of ∼140 kB. To minimize the risk of disruption of gene regulatory elements on chromosome 1, we chose to silence IL-24 expression by transfection with *Il24*-specific siRNAs as an alternative method. Compared with the introduction of control siRNA, IL-24 knockdown by siRNA also resulted in reduced expression of IL-10 in Th17 cells (Fig. 4 c).

Because we used APC-free systems to differentiate Th17 cells, we hypothesized that T cell–derived IL-24 had an autocrine or paracrine effect to promote the production of IL-10 in effector T cells in a feedback loop. To test this possibility, we used *Il20rb*^−/−^ T cells that cannot respond to extrinsic (soluble) IL-24. Notably, we found that *Il20rb*^−/−^ Th17 cells were not impaired in their capacity to produce IL-10, and thus did not phenocopy the properties of *Il24*^−/−^ Th17 cells (Fig. 4, d and e), suggesting that IL-24 exerted an effect on Th17 cells independent of its plasma membrane receptor. Also, addition of exogenous IL-24 did not rescue the lack of IL-10 production in *Il24*^−/−^ T cells under Th17 culture conditions, and conversely, blockade of IL-24 with neutralizing antibodies did not reduce IL-10 production in wild-type Th17 cells (Fig. 4 f), indicating that IL-24 subserved a robustly intrinsic effect on the regulation of IL-10 in Th17 cells that was independent of the surface expression of the IL-24 receptor on the plasma membrane.

To identify the failure of IL-24–deficient Th17 cells to produce IL-10 as an autonomous property of *Il24*^−/−^ T cells, we set up mixed cultures of IL-24–deficient and congenically marked wild-type T cells. Upon differentiation of naive sorted T cells into Th17 cells with TGF-β plus IL-6, *Il24*^−/−^ T cells always failed to express *Il10*, while their cocultured wild-type counterparts showed a robust amount of *Il10* mRNA (Fig. 4 g). Even naive *Il24*^−/−^ T cells that matured in an IL-24–sufficient environment because they grew up in mixed bone marrow chimeric (MBMC) mice with congenically marked wild-type plus *Il24*^−/−^ bone marrow showed the same behavior, i.e., IL-24–deficient Th17 cells essentially lacked IL-10 production, while their cocultured wild-type bone marrow–derived companion T cells showed robust induction of IL-10 (Fig. 4 g). Taken together, these data indicate that IL-24 acted by inducing IL-10 during Th17 differentiation in a cell-intrinsic manner.

### IL-24 maintains IL-10 production in Th17 cells in vivo

**Figure 5.**
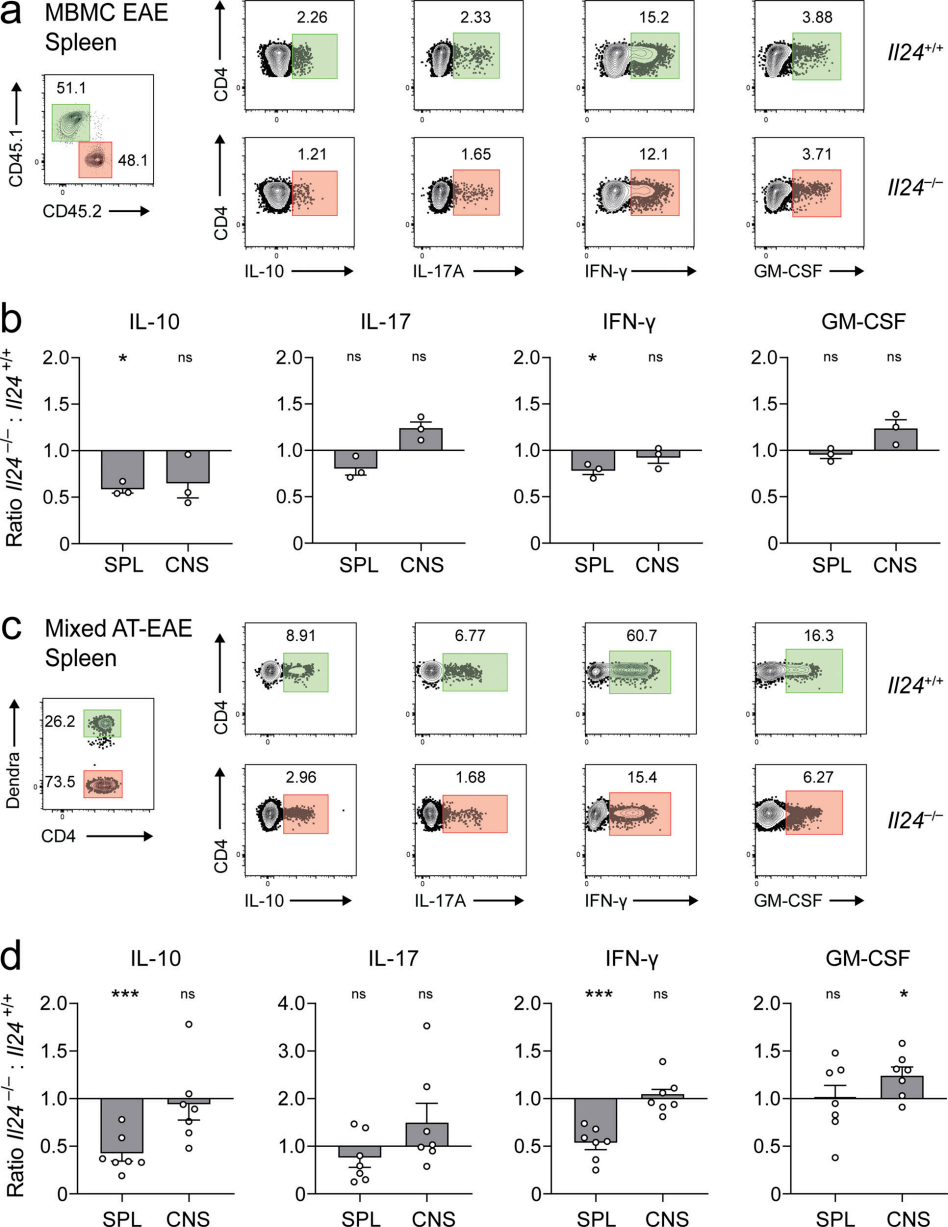
**IL-24 regulates IL-10 in Th17 cells in a cell-autonomous manner in vivo. (a and b)** MBMC mice were generated with a 1:1 chimerism of congenically marked CD45.1 wild-type (green gates) and *Il24*^−/−^ CD45.2 (red gates) bone marrow. **(a)** After induction of EAE, T cells were isolated at peak disease from the spleen (representative FACS plots, pregated for viable CD4^+^ cells) and the CNS (Fig. S2 a) and assessed for the production of cytokines by intracellular cytokine staining after ex vivo PMA/ionomycin stimulation. **(b)** Ratios of cytokine-positive fractions in IL-24–deficient vs. IL-24–sufficient T cells, summarized (mean ± SEM) from two independent experiments. **(c and d)** Mixed adoptive transfer EAE. Th17 cells were generated from naive wild-type 2D2 T cells (that were genetically labeled with mitoDendra2, a green fluorescent protein) or unlabeled naive IL-24–deficient 2D2 T cells. For cytokine production of these Th17 cells before transfer, see Fig. S2 b. **(c)** After cotransfer of wild-type (green gates) and IL-24–deficient 2D2 Th17 cells (red gates) into *Rag1*^−/−^ mice, T cells were reisolated from the spleen (pregated on viable CD4^+^ cells) and the CNS (Fig. S2 c) at the peak of EAE and subjected to intracellular cytokine staining. **(d)** Ratios of cytokine-positive fractions in IL-24–deficient vs. IL-24–sufficient 2D2 Th17 cells (seven biological replicates, mean ± SEM) after reisolation from host mice. In panels b and d, asterisks indicate significance level of one-sample *t* tests against a value of 1 (i.e., significantly different from an equal production of the respective cytokine in both compartments; ***, P < 0.001; *, P < 0.05).

**Figure S2.**
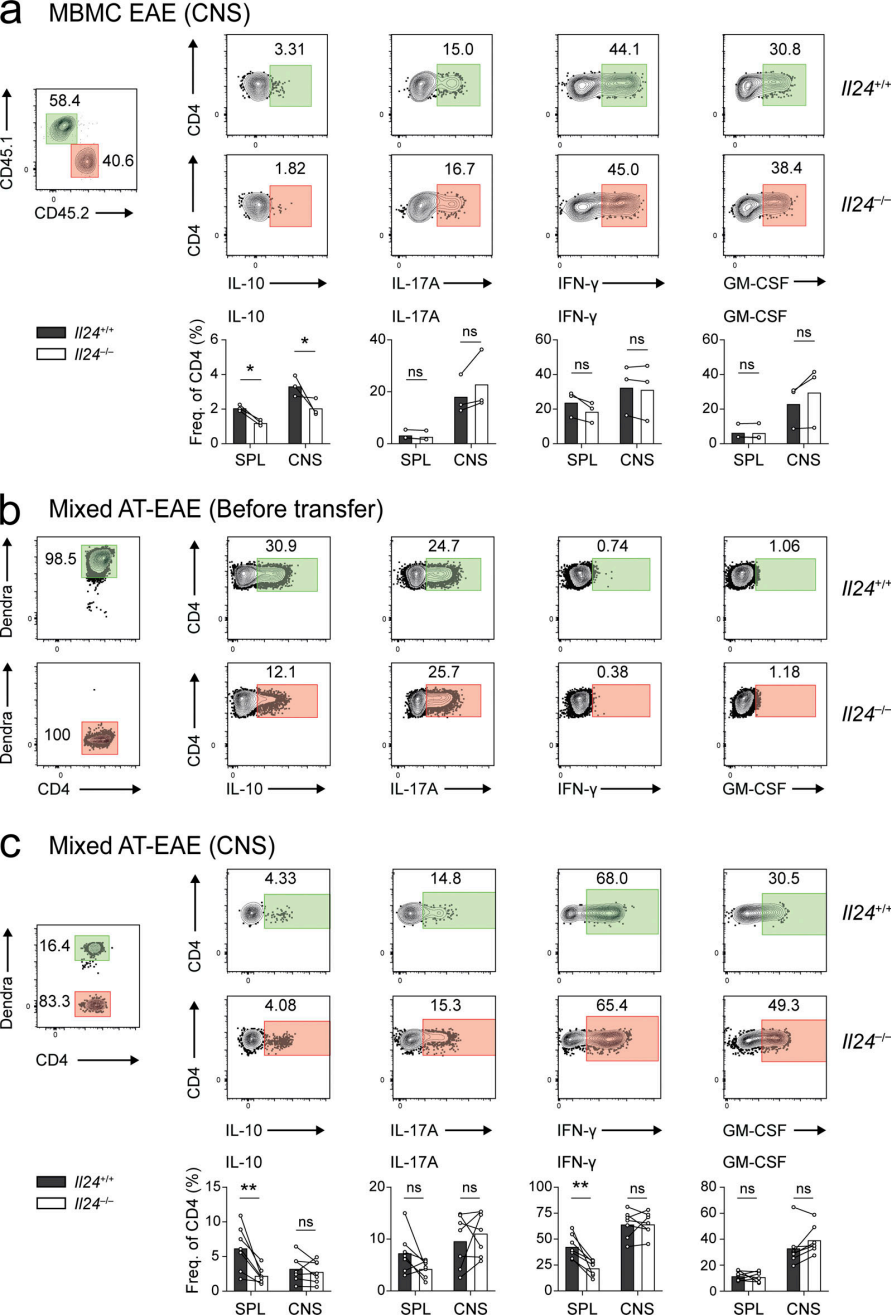
**IL-24 acts on T cells in a cell-autonomous manner. (a)** Cytokine production in congenically marked wild-type (CD45.1, green gates) or *Il24*^−/−^ (CD45.2, red gates) T cells (pregated on viable CD4^+^ cells) isolated from the CNS of mixed bone marrow chimeras at the peak of actively induced EAE; representative plots (upper panel) and quantification (lower panel, mean ± SEM of three mice) from two independent experiments. Asterisks indicate significance level (*, P < 0.05) of Holm–Sidak’s multiple comparison test after two-way ANOVA. **(b and c)** Cytokine staining in 2D2 Th17 cells used in the mixed adoptive transfer EAE model. Naive 2D2 T cells from wild-type mice (mitoDendra2^+^, green gates) and naive IL-24–deficient 2D2 T cells (red gates) were differentiated into Th17 cells. Intracellular cytokine staining in wild-type 2D2 Th17 cells (mitoDendra2^+^) and IL-24–deficient 2D2 Th17 cells before mixing and transfer into *Rag1*^−/−^ host mice; representative plots, pregated on viable CD4^+^ cells (b). Intracellular staining (likewise pregated on viable CD4^+^ cells) of wild-type 2D2 Th17 cells (mitoDendra2^+^, green gates) and IL-24–deficient 2D2 Th17 cells (red gates) after reisolation from the CNS of host mice at the peak of EAE (c); representative plots (upper panel) and quantification (lower panel, symbols depict individual mice). Asterisks indicate significance level (**, P < 0.01) of Holm–Sidak’s multiple comparison test after two-way ANOVA.

To test a potential T cell–autonomous effect of IL-24 in vivo, we used two complementary approaches. First, we immunized MBMC mice (wild-type plus *Il24*^−/−^ bone marrow) for EAE and found that IL-10 was reduced in the IL-24–deficient T cell compartment, while IL-10 was robustly induced in wild-type T cells in the spleen and also in the CNS of diseased mice around the peak of EAE (Fig. 5, a and b; and Fig. S2 a). Second, we performed a “mixed” adoptive transfer EAE. We cotransferred genetically labeled wild-type MOG T cell receptor transgenic (2D2) Th17 cells with IL-24–deficient 2D2 Th17 cells into recipient *Rag1*^−/−^ mice. Before transfer, IL-24–deficient 2D2 Th17 cells produced markedly less IL-10, as expected (Fig. S2 b). After re-isolation of the transferred T cells at the peak of EAE, IL-24–deficient 2D2 T cells maintained their dramatically decreased expression of IL-10, in particular in the spleen. In the CNS, this was less pronounced. Conversely, the GM-CSF expression in IL-24–deficient 2D2 T cells isolated from the CNS was significantly higher than in CNS-residing wild-type 2D2 T cells, consistent with prior reports (Chong et al., 2020; Fig. 5, c and d; and Fig. S2 c). In summary, these data lent credit to the idea that IL-24 promoted the induction and maintenance of IL-10 in Th17 cells in a cell-autonomous manner—a process that appeared universal but might be subject to a certain degree of compartment-specific modulation in vivo.

### An intracellular mode of action of IL-24 is sufficient to modulate IL-10 expression

**Figure 6.**
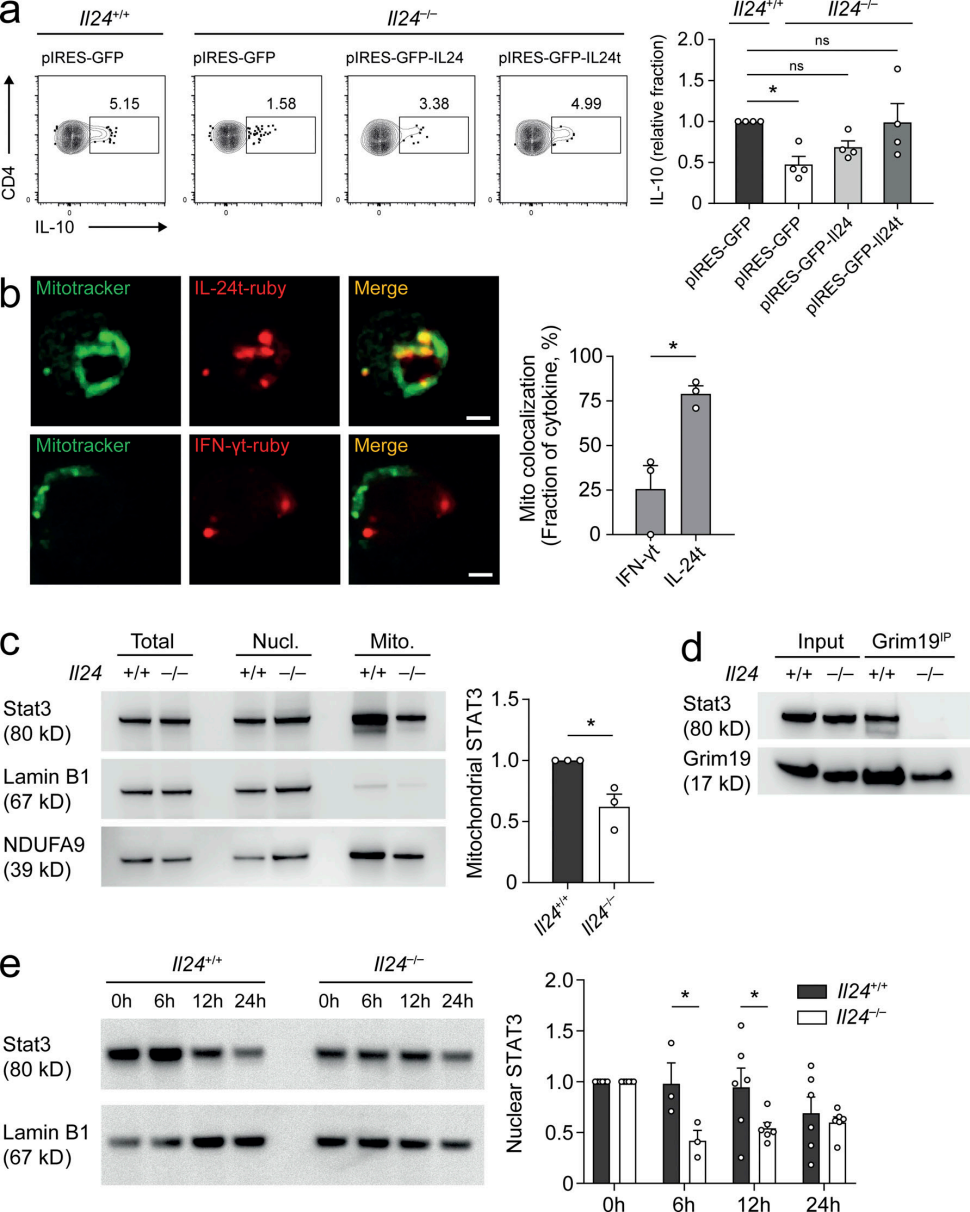
**Intracellular IL-24 localizes to the inner mitochondrial membrane and is necessary and sufficient for IL-10 production by Th17 cells. (a)** Naive T cells from *Il24*^+/+^ wild-type or *Il24*^−/−^ mice were cultured in Th17 differentiation conditions for 3 d. Th17 cells were nucleofected with pIRES-eGFP, pIRES-eGFP-IL-24, or pIRES-eGFP-IL-24t on day 1 and analyzed for intracellular IL-10 on day 3 with representative FACS plots (left, pregated for viable CD4^+^ GFP^+^ cells) and summary of four independent experiments (mean ± SEM) normalized to wild type (right). Relative fraction indicates IL-10^+^ frequency (among CD4^+^ cells) for the respective nucleofection, divided by the corresponding IL-10^+^ frequency of the *Il24*^+/+^ pIRES-eGFP nucleofected (control) cells for each experiment. Asterisks indicate significance level of Dunnett’s multiple comparison test after one-way ANOVA (*, P < 0.05). **(b)** Naive T cells from wild-type mice were cultured in Th17 differentiation conditions. On day 2 of differentiation, cells were nucleofected with mRuby2-N1-IL-24t (top row) or mRuby2-N1-IFN-γt (bottom row) and analyzed for intracellular cytokine distribution by confocal microscopy. Mitochondria were marked with Mitotracker. Representative microphotographs; scale bar, 2 μm (left). Quantification of the cytokine signal in the mitochondrial compartment (right) from three independent experiments (mean ± SEM). Asterisk indicates statistical significance of a two-tailed *t* test. **(c)** Naive wild-type or *Il24*^−/−^ T cells mice were differentiated into Th17 cells. On day 3, mitochondrial (Mito.) and nuclear (Nucl.) fractions were isolated and probed for STAT3. Lamin B1 and NDUFA9 were used as loading controls for nuclear and mitochondrial fractions, respectively (left). Densitometric quantification (right) of three independent experiments (mean ± SEM). Asterisk indicates statistical significance of a two-tailed *t* test. **(d)** Grim19 interacts with STAT3 in an IL-24–dependent manner. Naive T cells from wild-type or *Il24*^−/−^ mice were differentiated into Th17 cells. On day 3, cells were transferred into medium containing IL-2 (2 ng/ml). After 24 h, mitochondrial fractions were isolated, solubilized, immunoprecipitated with anti-Grim19 monoclonal antibody (Grim19^IP^), and probed for the amount of pulled STAT3 by Western blot. Solubilized mitochondrial fraction (input) was used as a control for coimmunoprecipitation. Representative Western blots from three independent experiments. **(e)** Loss of STAT3 from nuclear compartment. Naive T cells from wild-type or *Il24*^−/−^ mice were cultured in Th17 differentiation conditions for 3 d. After 3 d, cells were transferred into IL-2 medium (2 ng/ml) and analyzed for STAT3 in the nuclear compartment at baseline and after 6, 12, and 24 h. Representative Western blots (left) with normalized densitometry data (right) quantified from three to six independent experiments (mean ± SEM). Asterisks indicate significance level of Sidak’s multiple comparison test following two-way ANOVA (*, P < 0.05). Source data are available for this figure: SourceData F6.

IL-24 can act back on T cells via its surface receptor to modulate GM-CSF expression (Chong et al., 2020). However, IL-24 has also been linked with an intracellular function in previous studies (Sauane et al., 2004). To test this possibility during Th17 differentiation, we reconstituted *Il24*^−/−^ T cells with either wild-type IL-24 or a truncated version of IL-24 that could not be secreted and analyzed IL-10 production in T cells transduced with the different constructs after Th17 differentiation. Whereas *Il24*^−/−^ Th17 cells transduced with empty vector (GFP) failed to produce IL-10 as before, the expression of the nonsecretable version of IL-24 rescued their IL-10 production, and expression of secretable IL-24 resulted in a partial rescue of IL-10 in transduced Th17 cells (Fig. 6 a), demonstrating that intracellular IL-24 was sufficient to support IL-10 production in Th17 cells. Together, these data were consistent with the idea that a posttranscriptional, merely intracellular, function of IL-24 was sufficient to maintain the full capacity of Th17 cells to produce IL-10.

### IL-24 facilitates the relocation of STAT3 to mitochondria

Interestingly, IL-24 was reported to physically interact with Grim19 (Hu et al., 2016). Grim19 is an integral membrane protein located in the inner mitochondrial membrane and part of complex I of the respiratory chain (Fearnley et al., 2001). We hypothesized that intracellular IL-24 would also locate to the inner mitochondrial membrane. Therefore, a Flag-tagged construct of IL-24 was nucleofected into *Il24*^−/−^ T cells and assessed for its localization by confocal microscopy under Th17 conditions. A punctate cytosolic distribution of IL-24 was observed colocalizing with mitochondria as visualized by Mitotracker dye (Fig. 6 b). Grim19 has also been shown to interact with STAT3 and sequester STAT3 to the inner mitochondrial membrane (Tammineni et al., 2013). We hypothesized that the interaction between Grim19 and STAT3 was facilitated by IL-24. Therefore, we measured the amount of STAT3 channeled into the inner mitochondrial membrane in Th17 cells in the presence or absence of IL-24. *Il24*^−/−^ Th17 cells had less mitochondrial STAT3 than wild-type Th17 cells (Fig. 6 c), and STAT3 was indeed pulled by anti-Grim19 in wild-type but not in *Il24*^−/−^ Th17 cells (Fig. 6 d), suggesting that IL-24 mediated the Grim19-driven recruitment of STAT3 to the inner mitochondrial membrane in Th17 cells. To assess whether the different mitochondrial STAT3 pool sizes had an impact on the availability of nuclear STAT3 in wild-type vs. *Il24*^−/−^ Th17 cells, we analyzed the “decay” kinetics of nuclear STAT3 in Th17 cells after switching to IL-2 maintenance medium. Within 24 h, the decline of nuclear STAT3 was faster in *Il24*^−/−^ Th17 cells than in wild-type Th17 cells (Fig. 6 e). These data indicated that IL-24 indirectly affected STAT3 kinetics in the nucleus.

**Figure 7.**
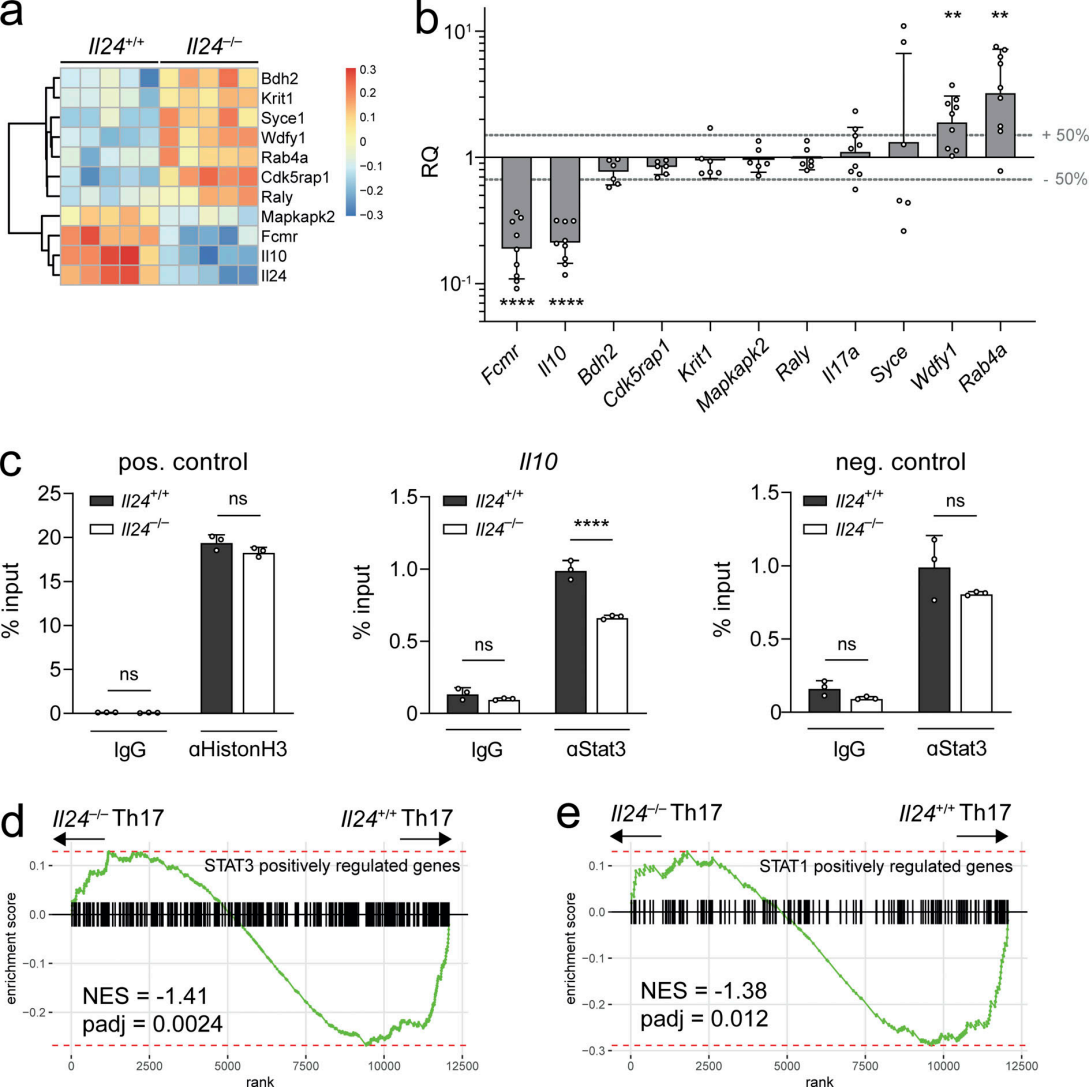
**Gene regulation in Th17 cells by IL-24. (a)** Heatmap with DESeq2-determined differentially expressed genes from five parallel cultures of wild-type or *Il24*^−/−^ Th17 cells subjected to bulk RNA-seq analysis. Naive T cells (CD3e^+^CD4^+^CD44^−^CD62L^+^CD25^−^) were isolated from splenocytes and lymph nodes using FACS and cultured for 3 d under Th17 skewing conditions (2 ng/ml TGFβ, 50 ng/ml IL-6, with 2 µg/ml soluble αCD28 and 4 µg/ml plate-bound αCD3). **(b)** Validation of RNA-seq candidate genes and *Il17* in RT-qPCR using TaqMan probes. Expression in *Il24*^−/−^ Th17 cells relative to wild-type Th17 cells (normalized to 1). Pooled relative quantification from *n* = 6–9 independent experiments (geometric mean ± geometric SD). Geometric mean values with at least 50% differential expression were tested using one-sample *t* tests against a value of 1. Asterisks indicate significance level (****, P < 0.0001; **, P < 0.01). **(c)** Results of ChIP-PCR. Naive T cells from *Il24*^+/+^ and *Il24*^−/−^ mice were cultured for 3 d under Th17 conditions, fixed, lysed, and chromatin fragmented for chromosome immunoprecipitation with anti-Histon H3 (positive control), anti-Stat3, or IgG control, followed by analysis of recovered DNA fragments by qPCR. For the positive control, a region in the *Rpl30* gene was amplified; for Stat3 pulling, either a region close to the intronic Stat-responsive element of the *Il10* gene or an unrelated gene desert on chromosome 14 were amplified (negative control). Asterisks indicate statistical significance of Sidak’s multiple comparison test (****, P < 0.0001) for three technical replicates (mean ± SD). **(d and e)** GSEA in *Il24*^−/−^ Th17 cells vs. wild-type Th17 cells. The transcriptomes of *Il24*^−/−^ Th17 cells vs. wild-type Th17 cells were probed for the enrichment of the IL-6–dependent STAT3 target gene set (Hirahara et al., 2015; d) and the IL-6–dependent STAT1 target gene set (Hirahara et al., 2015; e). NES, normalized enrichment score.

To test whether STAT3 target genes were dysregulated on a genome-wide level in *Il24*^−/−^ Th17 cells, we performed RNA-seq experiments in wild-type and IL-24 deficient Th17 cells differentiated with TGF-β plus IL-6. Overall, the transcriptomes of wild-type and *Il24*^−/−^ Th17 cells were very similar, and only few genes were differentially expressed, including *Il10*, *Fcmr*, *Rab4a*, and *Wdfy1* (Fig. 7, a and b). Classic STAT3 target genes including Socs3 were not regulated in response to genetic ablation of *Il24*, suggesting that the faster redistribution of STAT3 from the nuclear compartment in *Il24*^−/−^ Th17 cells was not associated with a global STAT3 phenotype. However, when we tested the loading of the *Il10* locus with STAT3 by ChIP-PCR, we found decreased amounts of STAT3 at the *Il10* locus of *Il24*^−/−^ Th17 cells compared with wild-type Th17 cells (Fig. 7 c). These data suggested that a certain degree of STAT3 dysregulation might still occur in *Il24*^−/−^ Th17 cells. Therefore, we interrogated the transcriptomes of wild-type vs. IL-24–deficient Th17 cells for STAT3 and STAT1 targets upon IL-6 stimulation, the balance of which has been considered as a determinant of the cytokine phenotype of Th17 cells (including IL-10 expression; Hirahara et al., 2015). We found that both STAT3 targets but also STAT1 targets were relatively enriched in wild-type vs. *Il24*^−/−^ Th17 cells (Fig. 7, d and e).

Altogether, the reduced surge of STAT3 and instead a persistent, less deflected presence of STAT3 in the nucleus in wild-type Th17 cells, in which IL-24 confers a STAT3 rheostat function to the inner mitochondrial membrane, may in addition increase the availability of STAT1 to be loaded on bona fide STAT1 target sites in the nucleus, a scenario that remains to be determined by biochemical analyses.

### IL-24 expression in T cells controls immunopathology in CNS autoimmunity

**Figure 8.**
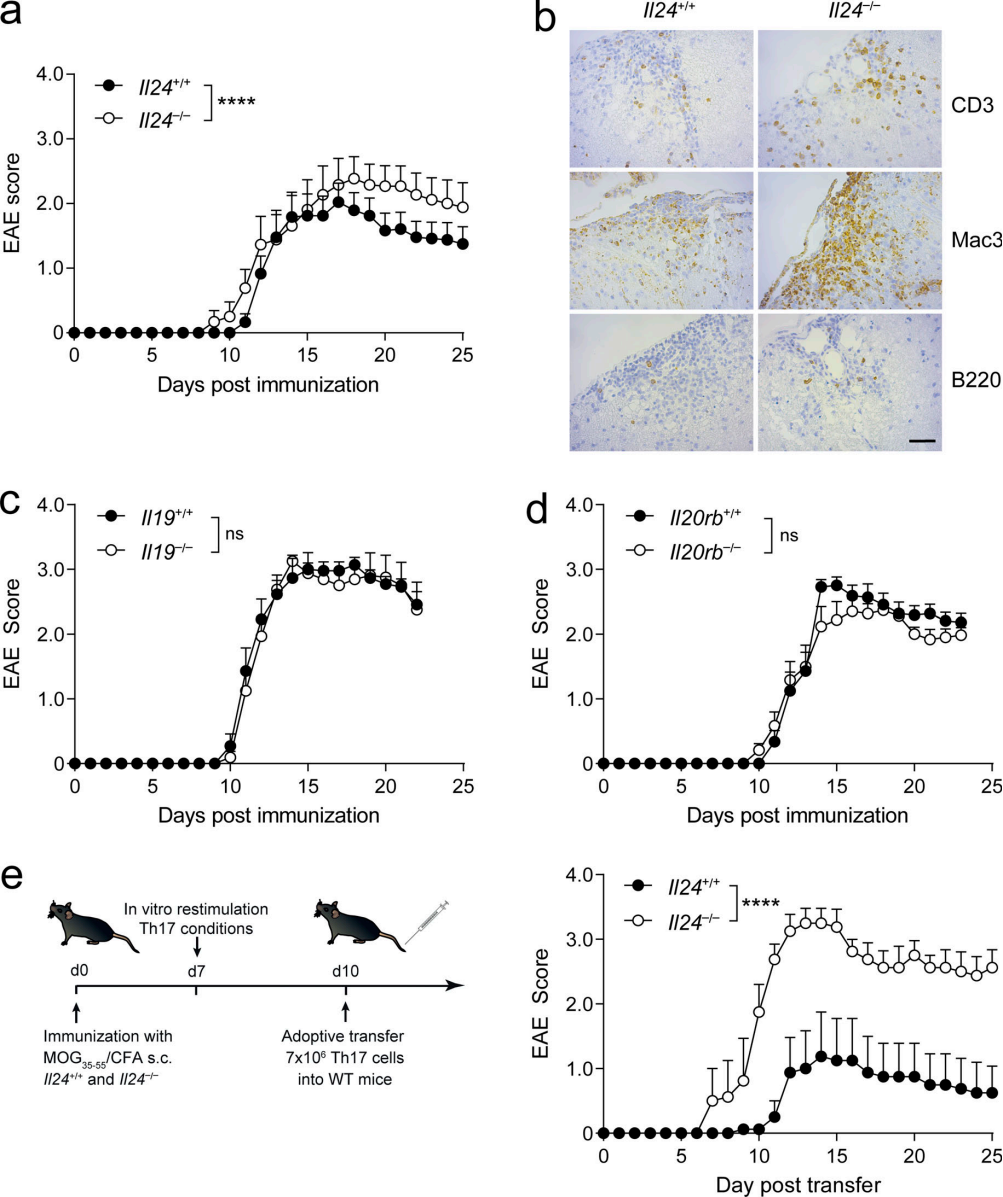
**Exacerbation of Th17 cell–dependent autoimmunity in the absence of IL-24 in T cells. (a)** *Il24*^+/+^ and *Il24*^−/−^ mice were immunized with an active EAE regimen, and disease severity was assessed daily. Representative disease course with ≥12 biological replicates (mean ± SEM) per group. **(b)** Immunohistochemistry of brain sections from EAE diseased *Il24*^+/+^ and *Il24*^−/−^ mice stained for CD3, B220, and Mac3. Representative sections; scale bar, 20 μm. **(c)** Representative EAE disease course of actively immunized *Il19*^+/+^ and *Il19*^−/−^ mice with at least eight biological replicates (mean ± SEM) per group. **(d)** Representative EAE disease course of actively immunized *Il20rb*^+/+^ and *Il20rb*^−/−^ mice with ≥14 biological replicates (mean ± SEM) per group. **(e)** Representative adoptive transfer EAE disease course of wild-type mice (right) after intravenous transfer of 7 × 10^6^ in vitro reactivated and Th17-skewed T cells from previously MOG/CFA-immunized *Il24*^+/+^ and *Il24*^−/−^ donor mice, according to the depicted procedure (left) with four biological replicates (mean ± SEM) per group. In panels a and c–e, asterisks indicate significance level from comparing slopes of origin-constrained linear regressions through the indicated disease courses using two-tailed unpaired *t* tests (****, P < 0.0001).

**Figure S3.**
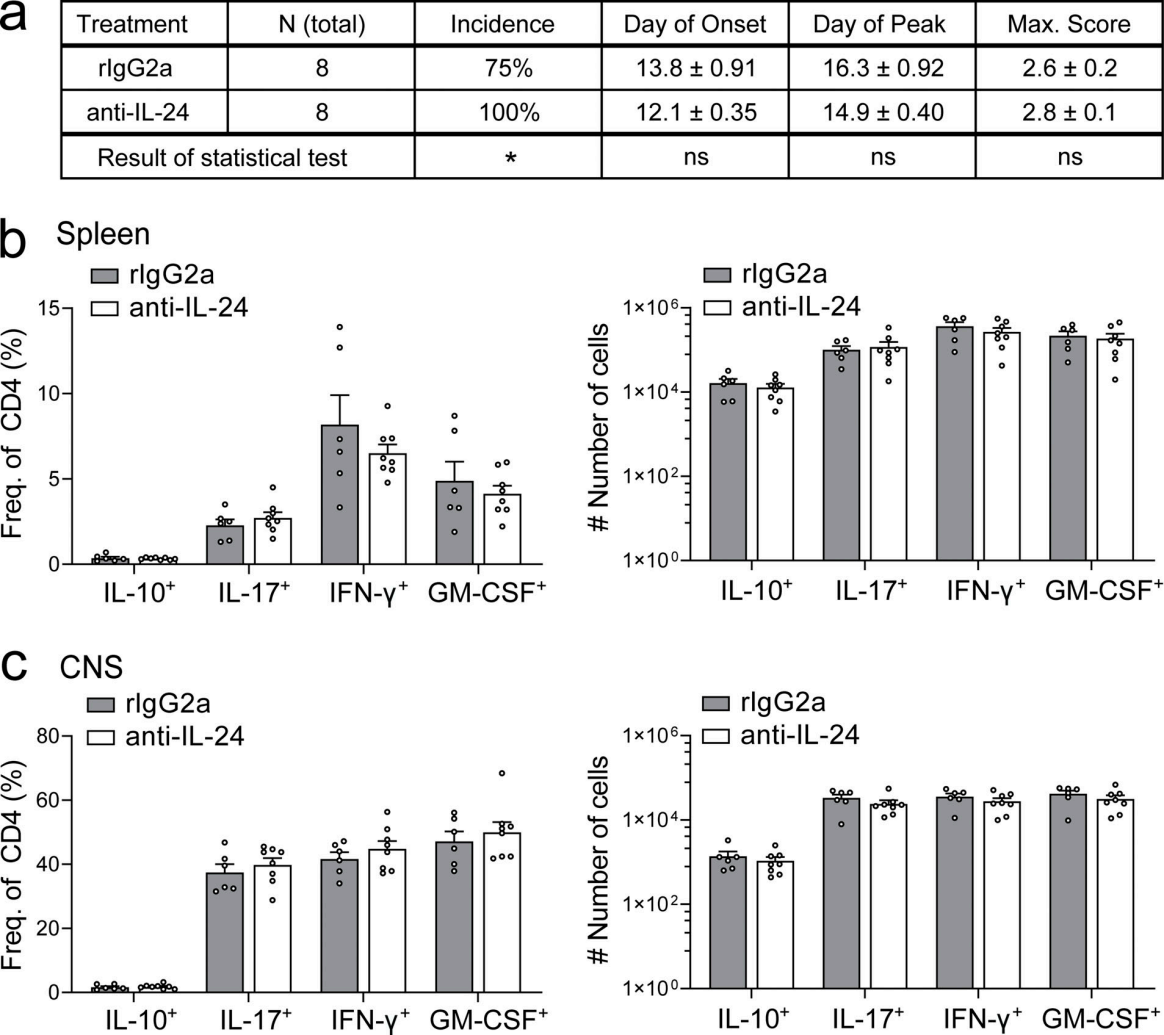
**Neutralization of IL-24 by monoclonal antibody fails to reduce IL-10 expression in T cells in vivo.** EAE was induced in wild-type C57BL/6 mice that were then treated with 100 µg i.p. of either control antibody (IgG2a) or anti–IL-24 every other day as of day 8 after immunization. **(a)** Characterization of the disease features with the results of statistical tests annotated (*, P < 0.05 in a log-rank test comparing incidence curves; two-tailed *t* tests for day of onset, day of peak and maximum score; ns, nonsignificant). **(b and c)** Fraction (left) and absolute number (right) of cytokine expressing CD4^+^ T cells in the spleen (b) and the CNS (c) at day 20 after immunization, with at least six biological replicates per group (mean ± SEM). Absence of statistical significance for all bars tested using two-way ANOVA followed by Sidak’s multiple comparison test.

Eventually, we wondered whether IL-24–dependent intrinsic regulation of IL-10 in Th17 cells produced a clinical phenotype. First, we induced EAE in wild-type control and *Il24*^−/−^ mice by immunization with MOG(35–55). Although disease onset was identical between groups, IL-24–deficient mice developed a more severe disease course (Fig. 8 a) that was reflected in a more intense inflammatory infiltrate in *Il24*^−/−^ mice compared with wild-type littermates (Fig. 8 b). Notably, genetic ablation of *Il19*, a cytokine closely related to *Il24* that also uses the same receptor complex, did not result in enhanced EAE severity (Fig. 8 c). Moreover, *Il20rb*^−/−^ mice did not show altered severity of EAE (Fig. 8 d), again suggesting that engagement of the key receptor for IL-20 family cytokines was irrelevant for the modulation of disease severity by IL-24. In addition, we performed an EAE experiment in wild-type mice that were either control-treated with IgG2a or with a monoclonal antibody to IL-24 as of day 8 after immunization. While we observed a slightly increased EAE incidence in the anti–IL-24–treated mice, the disease severity was similar in the control-treated and anti–IL-24–treated groups (Fig. S3 a). Also, the fraction and absolute number of IL-10–producing T cells in the spleen and CNS of both treatment groups was similar (Fig. S3, b and c), corroborating that the IL-10 phenotype of IL-24–expressing T cells may be a cell-autonomous feature that is not dependent on secreted IL-24.

Finally, since IL-24 is also produced by cells of ectodermal origin (Chen et al., 2018), we wanted to confirm that the clinical phenotype that we observed is due to lack of IL-24 in T cells. Therefore, we performed an adoptive transfer experiment where we primed encephalitogenic (MOG-specific) T cells in vivo in either wild-type or IL-24–deficient donor mice and—after restimulation in vitro—transferred equal numbers of activated CD4^+^ T cells into secondary wild-type hosts. While wild-type T cells induced a moderate disease in recipient mice (according to the titrated number of transferred T cells), the same number of *Il24*^−/−^ T cells generated a significantly more severe disease in secondary host mice (Fig. 8 e), supporting the immunomodulatory function of IL-24 in vivo in a model of Th17 cell–dependent immunopathology.

## Discussion

In this study, we provide evidence that IL-24 serves as a cell-intrinsic negative feedback regulator of Th17 cells. IL-24 is produced by Th17 cells independently of STAT3 activation but promotes the expression of IL-10 in Th17 cells at least in part through a STAT3-dependent mechanism. The IL-24–mediated induction of IL-10 in Th17 cells is independent of the cell surface receptor of IL-24. Instead, cytosolic IL-24 is shuttled to the mitochondria and facilitates the recruitment of STAT3 to the inner mitochondrial membrane. This creates a sink for STAT3 and results in a dampened (but sustained) availability of STAT3 in the nuclear compartment of Th17 cells. Therefore, we propose that Th17 cell–intrinsic IL-24 builds a rheostat for the nuclear translocation of STAT3, promoting its prolonged availability in the nucleus at the expense of surge-like STAT3 nuclear translocation events.

Effector cell–intrinsic autoregulation is a long-known theme in Th cell responses (Jankovic et al., 2010; Sher et al., 1991). This has perhaps been best worked out for Th1 cells, which start producing IL-10 upon sustained stimulation with IL-12 or in response to IL-27 (Saraiva et al., 2009; Stumhofer et al., 2007). In Th1 cell responses, the Th1-intrinsic production of IL-10 is an essential mechanism to prevent exaggerated immunopathology and cannot be compensated by Treg cells (Jankovic et al., 2007). We have reported that Th1/Th17 cells also respond to IL-12 and IL-27 with the production of IL-10 (Heinemann et al., 2014), but no similar feedback control loop has yet been described for bona fide Th17 cells. Here, we observed that IL-24, which is produced by Th17 cells, was associated with the capacity of Th17 cells to secrete IL-10. The induction of IL-24 in T cells either was dependent on TGF-β plus an additional signal (mediated by STAT3 or STAT1) or—in the absence of TGF-β such as in a Th17path differentiation scenario—depended entirely on STAT3 signal transduction, while STAT4 (induced by IL-12) was unable to drive IL-24 expression in T cells.

Recently, it has been suggested that IL-17 produced by Th17 cells might act back on Th17 cells to induce IL-24 in an NF-κB–dependent manner (Chong et al., 2020). IL-24, in turn, would bind its surface receptor on Th17 cells and—through induction of SOCS3—reduce the production of proinflammatory effector cytokines (including GM-CSF) by Th17 cells (Chong et al., 2020). Therefore, while STAT3 is required for the induction of IL-24, STAT3 is also downstream of the IL-24 receptor complex. However, we did not observe the IL-10–inducing effect of IL-24 in Th17 cells to be dependent on the expression of IL-20Rβ. Neither did we detect a consistent suppressive effect of IL-24 on the expression of IL-17 and GM-CSF (encoded by *Csf2*) in vitro. In fact, the regulation of *Il17* and *Csf2* are distinct, and IL-17 and GM-CSF are hardly coexpressed. Yet IL-24–deficient T cells tended to produce more GM-CSF in the CNS in vivo. Therefore, a directly suppressive effect of IL-24 on proinflammatory cytokines is possible (Chong et al., 2020). The effect of intracellular IL-24 on the regulation of *Il10* was specific and extremely robust both in vitro and in vivo. Interestingly, IL-1β, which has been associated with a “pathogenic” phenotype of Th17 cells (Ghoreschi et al., 2010), increased the amount of IL-24 in Th17 cells by stabilizing its mRNA. The stabilization of *Il24* mRNA is dependent on p38 (Otkjaer et al., 2010), and the stabilization of a variety of other mRNAs by IL-1β and IL-1α has been described (Dhamija et al., 2010; Sun and Ding, 2006). Therefore, similar to IL-12 for the induction of IL-10 in Th1 cells, IL-1β (even though it has been reported to suppress IL-10 transcription in Th17 cells; Zielinski et al., 2012) might eventually promote IL-10 secretion of Th17 cells through stabilizing IL-24. Because IL-1β is not produced by Th17 cells, this negative feedback loop is—just as for Th1 cells—dictated by T cell–extrinsic stimuli to allow for productive Th17 responses but limit exaggerated immunopathology.

An intracellular function of IL-24 has previously been suggested, since a nonsecretable version of IL-24 (also known as Mda-7) was found to be as efficient to induce apoptosis in a carcinoma cell line as wild-type IL-24 (Sauane et al., 2004). While the mode of action of intracellular IL-24 has not been investigated, IL-24 was found to bind to the NADH dehydrogenase (ubiquinone) 1 α subcomplex subunit 13 (Ndufa13, also known as Grim-19), which is a constituent of complex I of the respiratory chain in the inner mitochondrial membrane (Hu et al., 2016). Notably, Grim-19 is a chaperone to facilitate the import of STAT3 into the inner mitochondrial membrane and its integration into complex I (Tammineni et al., 2013). While the transcriptional effects of STAT3 are dependent on tyrosine phosphorylation and dimerization, the recruitment of STAT3 to the inner mitochondrial membrane requires serine phosphorylation (Reich, 2009). Our data suggest that IL-24 licenses Grim-19 for more efficient recruitment of STAT3 to the inner mitochondrial membrane. Due to the current model of a distinct function of mitochondrial STAT3 in enhancing oxidative phosphorylation (Wegrzyn et al., 2009), it is tempting to speculate that IL-24–deficient Th17 cells might exhibit reduced flux through the respiratory chain.

While Grim-19 produces a gain-of-function effect of STAT3 at the inner mitochondrial membrane, it dampens the transcriptional activity of STAT3 (Lufei et al., 2003; Zhang et al., 2003). Consistent with this concept, wild-type Th17 cells showed sustainedly reduced (but not abolished) amounts of STAT3 in the nucleus compared with *Il24*^−/−^ Th17 cells. STAT3 has a plethora of transcriptional targets in T cells (Hirahara et al., 2015), and the understanding how differential regulation of these targets is possible is limited (Murray, 2007). After removal of IL-6 as a STAT3-activating factor, we observed more sustained presence of STAT3 in the nucleus of wild-type than of IL-24–deficient T cells, and this behavior of STAT3 was associated with higher expression of IL-10 under Th17 differentiation conditions. It remains to be determined how this kinetics of STAT3 is linked to the regulation of IL-24 target genes, which are very selective compared with the large group of STAT3 target genes. In fact, although IL-24 recruits STAT3 to a “mitochondrial sink,” it appears that IL-24 is not a universal controller of STAT3-dependent genes.

*Il24* belongs to the *Il10* family of cytokines and is located in relative proximity to the *Il10* locus on chromosome 1. We consider it unlikely that the gene disruption strategy of *Il24* may have affected regulatory elements of *Il10* to result in reduced expression of IL-10. First, *Il19*—another gene of the *Il10* cluster with even closer proximity to *Il24*—was not affected in *Il24*^−/−^ mice. Second, acute editing of the *Il24* locus reproduced the phenotype of *Il24*^−/−^ T cells. Third, silencing of IL-24 by siRNA, which leaves the genomic *Il10* cluster unaffected, also reduced IL-10 expression in Th17 cells.

In summary, the IL-10 family cytokine IL-24, which serves an important role in physiologic and pathophysiologic responses of epithelia, may have been coopted by Th17 cells (key adaptive mediators of host defense at epithelial tissues) to control exaggerated Th17-mediated immunopathology in this anatomic niche. Further studies will have to test whether IL-24 can be exploited to control Th17-driven immunopathology in autoimmunity and perhaps cancer development induced by chronic inflammation.

## Materials and methods

### Animals

C57BL/6 wild-type mice, CD45.1 mice (B6.SJL-Ptprca Pepcb/BoyJ), *Il24*^−/−^ mice (B6N[Cg]-Il24^tm1.1(KOMP)Vlcg^/2J), *Rag1*^−/−^ (B6.129S7-Rag1^tm1Mom^/J), and VertX reporter mice (Il10^tm1.1Karp^) were obtained from the Jackson Laboratory. *Il19*^−/−^ mice (C57BL/6N-Il19^tm2e(EUCOMM)Hmgu^/H) were obtained from the European Mouse Mutant Archive at the MRC Harwell Mouse Genetics Research Institute. *Il20rb*^−/−^ mice (Il20rb^tm1Uwe^; Wahl et al., 2009) were obtained from the University of Ulm (courtesy of Franz Oswald). Cas9 mice (Gt[ROSA]26Sor^tm1(CAG-cas9^*^,-EGFP)Fezh^/J) were crossed with CD4-cre mice (Tg[Cd4-cre]1Cwi/BfluJ), both obtained from the Jackson Laboratory, to generate Cas9-CD4-cre mice. Smart17A/Great reporter mice (B6.129S4-Il17a^tm1.1Lky^ × C129S4[B6]-Ifng^tm3(EYFP)Lky^/J; Price et al., 2012) were obtained from the University of California, San Francisco (courtesy of Richard Locksley) and crossed with VertX reporter mice to generate triple-reporters. 2D2 × DendraGreen reporter mice were generated by crossing Pham^floxed^ reporter mice (B6;129S-Gt[ROSA]26Sor^tm1(CAG-COX8A/Dendra2)Dcc^/J) with CD4-Cre mice (B6.Cg-Tg[Cd4-cre]1Cwi/BfluJ) and 2D2 mice (C57BL/6-Tg[Tcra2D2,Tcrb2D2]1Kuch/J), all obtained from the Jackson Laboratory. 2D2 × *Il24*^−/−^ mice were generated by crossing *Il24*^−/−^ mice with 2D2 mice.

All mouse strains were on C57BL/6J background. Animals were kept in a specific pathogen–free facility at the Technical University of Munich with a dark/light cycle of 12 h, temperature of 20–24°C, and humidity of 45–60%. All experimental protocols were approved by the standing committee for experimentation with laboratory animals of the Bavarian state authorities and were carried out in accordance with the corresponding guidelines (ROB-55.2-2532.Vet_03-18-53 and ROB-55.2-2532.Vet_02-17-234). 8–12-wk-old age- and sex-matched mice were used for all experiments.

### Generation of mixed bone marrow chimeras

Recipient mice were irradiated at a dose of 11 Gy. A total of 10–20 × 10^6^ donor bone marrow cells mixed 1:1 from CD45.1 wild-type donors and *Il24*^−/−^ mice, depleted of CD90.2^+^ cells using Miltenyi Microbeads (130-121-278), were injected i.v. into recipients within 16–20 h after irradiation. The reconstituted mice were maintained on antibiotic water (0.1 mg/ml, enrofloxacin; Bayer) for 3 wk after transplantation. The reconstitution of the hematopoietic compartment was assessed 5–6 wk after cell transfer in peripheral blood.

### Induction of EAE

EAE was induced by subcutaneous immunization in the base of tail with 200 μg of MOG(35–55) peptide (MEVGWYRSPFSRVVHLYRNGK; Auspep) in CFA containing 500 μg *Mycobacterium tuberculosis* H37Ra (231141; BD Biosciences) per mouse plus i.v. or i.p. injection of 200 ng pertussis toxin (PTx, P7208-50UG; Sigma-Aldrich) on days 0 and 2 after immunization. Disease progression and severity were assessed as described before (Korn et al., 2007). The onset of disease was typically 11–13 d, and the peak of disease was typically 15–20 d after immunization. For the anti–IL-24 treatment EAE, wild-type mice were immunized as described above and injected i.p. every other day, starting on day 8, with 100 µg of rat IgG2a (BE0089; BioXcell) or anti–IL-24 (MAB2786; R&D Systems) dissolved in PBS.

### Adoptive transfer of T cells

For adoptive transfer EAE experiments into wild-type recipients, C57BL/6 wild-type donor mice were immunized with MOG(35–55) peptide in CFA and PTx according to the regimen for active induction of EAE. On day 7, draining lymph nodes and spleens were prepared, pooled, and restimulated ex vivo for 3 d with 35 µg/ml MOG(35–55) in the presence of TGF-β (0.25 ng/ml), IL-6 (5 ng/ml), IL-23 (6.5 ng/ml), and anti–IFN-γ (10 µg/ml) to skew antigen-specific T cells into Th17 cells. After isolation of CD4^+^ T cells from the recall culture using Miltenyi untouched CD4^+^ T cell purification beads, 7 × 10^6^ CD4^+^ T cells were transferred i.v. into recipient wild-type mice, concomitantly with i.p. injection of 200 ng PTx on days 0 and 2 after adoptive transfer. For mixed adoptive transfer EAE into *Rag1*^−/−^ recipients, naive T cells from 2D2 × DendraGreen reporter mice and 2D2 × *Il24*^−/−^ mice were isolated, polyclonally stimulated on anti-CD3–coated plates in Th17 conditions as outlined in the T cell cultures section, retrieved after 3 d, and then injected i.v. into *Rag1*^−/−^ mice, alongside i.p. injection of PTx on days 0 and 2 after adoptive transfer.

### Immunohistochemistry

Mice were sacrificed under deep anesthesia by intracardial perfusion with PBS followed by perfusion with 4% wt/vol paraformaldehyde dissolved in PBS. Brains were removed and fixed in 4% paraformaldehyde overnight. Vertebral columns including the spinal cords were additionally decalcified with Osteosoft (Sigma-Aldrich) for 72 h before paraffin embedding; 5-μm-thick sections were stained with H&E and Luxol-fast blue/periodic acid Schiff. Immunohistochemistry was performed with CD3 (1:50, C1597R0; DCS), MAC-3 (clone M3/84), and B220 (clone RA3-6B2; eBioscience). Images were analyzed using Aperio Image Scope (Leica) in a blinded manner.

### Preparation of mononuclear cells from the CNS

Mice were perfused through the left cardiac ventricle with ice-cold PBS. The brain was dissected, and the spinal cord was flushed out with PBS by hydrostatic pressure. CNS tissue was cut into pieces and digested with 1 mg/ml collagenase D (11088866001; Roche Diagnostics) and 40 μg/ml DNase I (04716728001; Roche Diagnostics) at 37°C for 30 min, passed through a 100-μm cell strainer (542000; Greiner Bio-One), and pelleted by gravity centrifugation (400 *g*, 4°C, 10 min), followed by a Percoll gradient (70%/37%, 17-0891-01; GE Healthcare) centrifugation (640 *g*, 20°C, 22 min). Mononuclear cells were isolated from the interphase, washed, and resuspended in FACS buffer (PBS with 2% FCS) for further analysis.

### Isolation of naive T lymphocytes and T cell cultures

Lymph nodes and spleens were passed through a 70-μm cell strainer (542070; Greiner Bio-One), followed by centrifugation (400 *g*, 4°C, 10 min). Spleen samples underwent erythrocyte lysis with BD Pharm Lyse (555899; BD Biosciences). Naive CD4 T cells were enriched using either magnetic bead separation (MACS; 130-104-453; Miltenyi) or FACS, sorted for CD4^+^ (1:100; eBioscience), CD25^−^ (1:50; eBioscience), CD62L^+^ (1:100; BD Biosciences), and CD44^−^ (1:100; BD Biosciences). T cells were cultured for 3 d with soluble anti-CD28 (2 µg/ml, BE0001-1; BioXCell) on cell culture plates coated with anti-CD3 (4 µg/ml, BE0001-1; BioXCell) in complete DMEM as described (Hiltensperger et al., 2021).

T cells were skewed toward Th subsets by culturing in the presence of 10 ng/ml IL-12 (419-ML; R&D Systems) and 10 µg/ml anti-IL4 (BE0045; BioXCell) for Th1 cells (with transfer to uncoated plates after 1 d); 20 ng/ml IL-4 (130-097-761; Miltenyi) and 10 µg/ml anti–IL-12 p40 (BE0051; BioXCell) for Th2 cells; 2.5 ng/ml TGF-β (130-095-067; Miltenyi) for iTreg cells; 100 ng/ml IL-27 (2799-ML; BioXCell) with or without 2.5 ng/ml TGF-β for Tr-1 cells; 50 ng/ml IL-6 (130-095-067; Miltenyi) and 2 ng/ml TGF-β for Th17 cells with or without 25 ng/ml IL-1β (130-101-681; Miltenyi); and 25 ng/ml IL-6, 25 ng/ml IL-23 (130-096-676; Miltenyi), and 25 ng/ml IL-1β for Th17path cells.

For coculture experiments, naive T cells from CD45.1 or *Il24*^−/−^ mice were mixed at a 1:1 ratio (or taken directly from 1:1 MBMC mice), cultured for 3 d, and separated using FACS with anti-CD45.1 (1:100; BioLegend) and anti-CD45.2 (1:100; Thermo Fisher Scientific) for subsequent analysis. For rescue experiments, recombinant human IL-24 (10 ng/ml, 1965-IL; R&D Systems) or mouse IL-24 (10 ng/ml, 7807-ML; R&D Systems) was added to the culture medium. For blocking experiments, anti–IL-24 antibodies (MAB2786 and AF2786; R&D Systems) or their respective isotype controls (rat IgG2a, BE0089; BioXcell; and goat IgG, AB-108C; R&D Systems) were added, all at 10 µg/ml. For Stat3 assessments, cells were cultured under Th17 conditions as stated above and then transferred into medium with IL-2 (2 ng/ml, 130-094-055; Miltenyi) for 24 h.

For proliferation assays, naive T cells were labeled with CellTrace Violet Proliferation Dye (Thermo Fisher Scientific) according to the manufacturer’s instructions. Dilution of proliferation dye was assessed after 3 d by flow cytometry, alongside cytokine staining. Proliferation index was calculated with R v3.6.3 (R Core Team, 2019) using the package flowFit v1.24.0.

### ELISA

Secretion of IL-24 was detected by standard sandwich ELISA (DuoSet; R&D Systems). Standard curves and sample concentrations were calculated based on the mean of triplicates for each dilution or sample.

### Intracellular cytokine staining and flow cytometry

Cells were stimulated with PMA (0.1 μg/ml; Sigma-Aldrich) and ionomycin (1 μg/ml; Sigma-Aldrich) in the presence of GolgiStop (1 μl/ml; BD Biosciences) for 2 h. The cells were harvested and incubated with anti-CD4 (1:100; BD Biosciences or BioLegend) in PBS containing Fc-block (1:100) and Live-Dead stain (1:500; Invitrogen). Subsequently, cells were washed and fixed with 2% formalin (Roti Histofix, Carl Roth) for 1 h at 4°C or with Cytofix/Cytoperm (BD Biosciences) for 20 min at 4°C. For intracellular staining, cells were incubated with anti–IL-10 (1:100; BD Biosciences), anti–IL-17A (1:100; BD Biosciences or BioLegend), anti–IFN-γ (1:100; eBioscience), anti–GM-CSF (1:100; BD Biosciences), and anti-Foxp3 (eBioscience) for 1 h at 4°C in permeabilization buffer (eBioscience) or Perm/Wash (BD Biosciences). The cells were washed twice and resuspended in FACS staining buffer. Cells from the Smart17A reporter mice were stained with anti-hNGFR (1:20; BioLegend) to detect IL-17–expressing cells in flow cytometry. Flow cytometric analysis was performed on a CytoFLEX flow cytometer (Beckman Coulter) with CytExpert (v.2.3.1.22) software or, for sorting, on a FACS Aria III machine (BD Biosciences) with BD FACSDIVA (v8.0.1) software, and flow cytometric data were analyzed using FlowJo (v10.5.1) software (BD Biosciences). Absolute CD4^+^ cell counts were determined using a Guava easyCyte 5HT cytometer (Merck) with anti-CD4 (1:100; BD Biosciences) and Live-Dead stain (1:500; Invitrogen).

### DNA constructs

The sequences corresponding to full-length or nonsecretable (truncated) versions of IL-24, i.e., IL-24 and IL-24t, were cloned into mammalian expression vector pIRES-eGFP (6029-1; Clontech). The fusion constructs of nonsecretable IL-24 (mRuby2-N1-IL-24t; sequence: 5′-ATG​CAA​GAG​TTC​CGA​TTT​GGG​TCT​TGC​CAA​GTG​ACA​GGG​GTG​GTT​CTC​CCA​GAA​CTG​TGG​GAG​GCC​TTC​TGG​ACT​GTG​AAG​AAC​ACT​GTG​CAA​ACT​CAG​GAT​GAC​ATC​ACA​AGC​ATC​CGG​CTG​TTG​AAG​CCG​CAG​GTT​CTG​CGG​AAT​GTC​TCG​GGT​GCT​GAG​AGC​TGT​TAC​CTT​GCC​CAC​AGC​CTG​CTG​AAG​TTC​TAC​TTG​AAC​ACT​GTT​TTC​AAG​AAC​TAC​CAC​AGC​AAA​ATA​GCC​AAA​TTC​AAG​GTC​TTG​AGG​TCA​TTC​TCC​ACT​CTG​GCC​AAC​AAC​TTC​ATA​GTC​ATC​ATG​TCA​CAA​CTA​CAG​CCC​AGT​AAG​GAC​AAT​TCC​ATG​CTT​CCC​ATT​AGT​GAG​AGT​GCA​CAC​CAG​CGG​TTT​TTG​CTG​TTC​CGC​AGA​GCA​TTC​AAA​CAG​TTG​GAT​ACA​GAA​GTC​GCT​TTG​GTG​AAA​GCC​TTT​GGG​GAA​GTG​GAC​ATT​CTC​CTG​ACC​TGG​ATG​CAG​AAA​TTC​TAC​CAT​CTC-3′) or IFN-γ (mRuby2-N1-IFN-γt; sequence: 5′-ATG​CAC​GGC​ACA​GTC​ATT​GAA​AGC​CTA​GAA​AGT​CTG​AAT​AAC​TAT​TTT​AAC​TCA​AGT​GGC​ATA​GAT​GTG​GAA​GAA​AAG​AGT​CTC​TTC​TTG​GAT​ATC​TGG​AGG​AAC​TGG​CAA​AAG​GAT​GGT​GAC​ATG​AAA​ATC​CTG​CAG​AGC​CAG​ATT​ATC​TCT​TTC​TAC​CTC​AGA​CTC​TTT​GAA​GTC​TTG​AAA​GAC​AAT​CAG​GCC​ATC​AGC​AAC​AAC​ATA​AGC​GTC​ATT​GAA​TCA​CAC​CTG​ATT​ACT​ACC​TTC​TTC​AGC​AAC​AGC​AAG​GCG​AAA​AAG​GAT​GCA​TTC​ATG​AGT​ATT​GCC​AAG​TTT​GAG​GTC​AAC​AAC​CCA​CAG​GTC​CAG​CGC​CAA​GCA​TTC​AAT​GAG​CTC​ATC​CGA​GTG​GTC​CAC​CAG​CTG​TTG​CCG​GAA​TCC​AGC​CTC​AGG​AAG​CGG​AAA​AGG​AGT​CGC​TGC-3′) were generated by subcloning into mRuby2-N1 (54614; Addgene). The gRNAs targeting IL-24 or GFP were cloned into a retroviral vector (pMSCV-U6-guide-IRES-Thy1.1) using Gibson’s assembly kit (E2611S; New England Biolabs).

The *Il24* minimal promoter sequence (Sahoo et al., 2011) used for luciferase assays was 5′-TCA​TCT​CAC​CTG​AGG​GAC​TGA​TTT​CTG​CCC​CCA​CCC​CCC​TGT​CTA​AGA​GCA​AAG​GGT​GAC​TAG​GTG​ATG​AAG​TAT​TTC​TCC​AGG​GAA​GCA​TGA​CCA​ATT​TCC​CTT​CCT​CCA​CAT​TCC​CCT​CCT​CTG​CCC​CTC​CCT​GCC​AGA​CCC​CTT​ATA​TAC​AGT​TCT​CCC​AGC​CTT​GCT​TAC​CCT​CAG​TCT​TTC​ACT​TTT​GAA​ATC​ATT​TCC​ACA​GCT​GAG​AAG​GAG​CTT​CCC​ACC​CAG​CAG​AAG​ATC​CTC​TAC​CAA​TGA-3′. This minimal promoter was subcloned in the multiple cloning site of the pXPG plasmid (71248; Addgene) by introducing SacI and HindIII binding sites during PCR amplification using modified primers.

The 3′ UTR sequence of *Il24* used for luciferase assays, with AU-rich regions highlighted in bold, was 5′-CTG​CTG​ATT​GGA​TAA​CTT​CCT​CCT​TTG​CTC​TCC​ATG​CCA​TTT​CAA​GGC​ATT​GTG​TAC​ATC​CCT​GCT​GTC​CTC​AAG​GCA​CTT​CAG​ACC​CTT​GGC​CAT​GGA​CCC​CGT​TGT​TGG​CTC​AGG​CTT​TTC​CTC​AGA​CCT​CAC​TCT​TCA​GTC​CAA​ATG​ACA​GCC​ATA​GAT​GGC​ACC​TTT​GGA​TGC​TCC​GAC​TGA​CCC​ACA​AAG​TAG​ATT​TGC(**ATA​TTT​ATT​A**)CAG​CCC​TAT​TAA​ATT​ATT​GTC​ACC​TTC​CCT​GGA​AAC​CGT(**ATT​TA**)TTT​GTG​AGA​CCA​GAA​GTT​CCA​TGA​AAG​CAT​CAG​A(**ATT​TA**)GTG​CCC​CAT​GCC​TCC​TCC​TCA​CTT​CCT​GTG​ATC​TGG​CTC​AGC​ATG​GGG​GCA​GTG​GAT​GGT​TGC​TCA​G(**TAA​ATA​TTT​AAA​AT**)GGA-3′. The sequence with or without AU-rich regions was subcloned into the multiple cloning site of pMIR-GLO Dual-Luciferase miRNA Target Expression Vector (E1330; Promega), 3′ of the firefly luciferase gene, by introducing SacI and SbfI binding sites during PCR amplification using modified primers.

### Retroviral transduction and transfection

#### Retroviral transduction

Retroviral constructs carrying gRNAs targeting GFP (MSCV-U6-GFPguide-IRES-Thy1.1) or IL-24 (MSCV-U6-IL-24guide-IRES-Thy1.1) were transfected into Platinum E cells (RV-101; Cell Biolabs) using Lipofectamine LTX reagent with PLUS reagent (15338100; Thermo Fisher Scientific). Naive T cells (CD4^+^CD44^−^) purified from CD4-Cre × Rosa26-LSL-Cas9 knock-in mice were differentiated into Th17 cells and, on day 1 of differentiation, were spinoculated with retrovirus as described previously (Heinemann et al., 2014). Briefly, the retroviral supernatant harvested on day 2 of transfection was centrifuged (3,000 *g*, 1.5 h, 32°C) and used for transduction by adding 0.5 ml on Th17 cells (10^5^) plated in 24-well tissue culture plates (1 ml/well) precoated with 0.1 µg/ml retronectin (T100B; Takara BioScience). Spin transduction was carried out by centrifugation at 32°C (800 *g*, 1.5 h). Transduced cells were identified using anti-Thy1.1 staining (1:100; BD Biosciences).

#### Nucleofection

MACS-sorted naive T cells (CD4^+^CD44^−^) were cultured in Th17 differentiation conditions for 3 d. Th17 cells (10^6^) were nucleofected with 5 µg of pIRES-eGFP, pIRES-eGFP-IL-24, pIRES-eGFP-IL-24t, mRuby2-N1-IL-24t, or mRuby2-N1-IFN-γt using Amaxa P3 Primary Cell 4D-Nucleofector X Kit L (V4XP-3024; Lonza) and program DN-100 (for mouse T cells) in a 4D-Nucleofector unit (Lonza) as per the manufacturer’s instructions.

#### RNA interference

Naive T cells purified from wild-type mice (by MACS) were differentiated into Th17 cells in reduced-serum medium (siRNA delivery medium; B005000-100; Dharmacon). On day 1, cells were treated with Accell siRNA oligos targeting IL-24 (1 µm, E-050687-00-0005; Dharmacon) or control oligos (1 µm, D-001910-01-05; Dharmacon). On day 3, cells were analyzed for intracellular IL-10 and IL-17A.

### Intracellular distribution and confocal microscopy

MACS-sorted naive T cells from wild-type mice were cultured in Th17 differentiation conditions. On day 1, T cells (10^6^) were nucleofected with 5 µg of mRuby2-N1-IL-24t or mRuby2-N1-IFN-γt and plated into poly-L-lysine–coated chamber slides (80824; Ibidi). On day 2, cells were treated with Mitotracker green (10 nm, M7514; Thermo Fisher Scientific) for 30 min. Cells were washed and analyzed by confocal microscopy. Images were acquired at 60× magnification using a Leica SP8 confocal microscope and Leica Applications Suite X (v3.5.6.21594) software. Cytokine signal colocalization was quantified using ImageJ (1.52i).

### Subcellular fractionation, immunoprecipitation, and Western blotting

Th17 cells (5–20 × 10^6^) were harvested and washed with ice-cold PBS. The mitochondrial or nuclear factions were isolated using a mitochondria isolation kit (89874; Thermo Fisher Scientific) according to the manufacturer’s protocol. For immunoprecipitation, the mitochondrial fraction was solubilized using non-denaturing lysis buffer (20 mM Tris HCl, 137 mM NaCl, 1% NP-40, and 2 mM EDTA, pH 8.0) containing 2% *n*-dodecyl-β-D-maltoside (89902; Thermo Fisher Scientific) at 4°C. Mitochondrial fractions (50–100 µg in 100 µl) were incubated with anti-Grim19 (5 μg) for 12 h at 4°C. The reaction mixture was incubated with 50 µl of protein-A/G Sepharose beads (Ab193262; Abcam) for 1 h at 4°C on a rotator. Subsequently, protein-A/G beads were washed, and immunoprecipitated proteins were eluted using SDS sample buffer. The samples were resolved on 10% NuPAGE gel (NP0302BOX; Thermo Fisher Scientific) and transferred onto PVDF membrane using the iBlot system (Thermo Fisher Scientific). The membranes were incubated with Super Block (37581; Thermo Fisher Scientific) for 1 h at room temperature to block nonspecific binding. Membranes were incubated with primary antibodies (diluted in Super Block) overnight at 4°C. After washing with PBST (PBS with 0.05% Tween 20), antibody binding was detected with HRP-conjugated secondary antibodies and SuperSignal west femto maximum sensitivity substrate (34095; Thermo Fisher Scientific).

### Quantitative RT-PCR (RT-qPCR)

Bulk RNA was extracted using RNeasy (Qiagen) or Zymo Direct-zol RNA Microprep columns (Zymo Research). cDNA was generated from mRNA using TaqMan Reverse Transcription reagents (Applied Biosystems) and used as template for quantitative PCR (qPCR). If not otherwise specified, TaqMan Assays (Thermo Fisher Scientific) were performed to analyze relative gene expression (*Il10*, Mm00439614_m1; *Il17a*, Mm00439618_m1; *Il24*, Mm00474102_m1; *Ifng*, Mm00801778_m1; *Bdh2*, Mm00459075_m1; *Cdk5rap1*, Mm00482296_m1; *Fcmr*, Mm01302388_m1; *Krit1*, Mm01316552_m1; *Mapkapk2*, Mm01288465_m1; *Rab4a*, Mm01253178_m1; *Raly*, Mm00499167_m1; *Syce*, Mm01279053_m1; and *Wdfy1*, Mm00840455_m1) using a OneStep Plus Real Time PCR System (Applied Biosystems). Gene expression was normalized to the expression of *Actb* (Mm02619580_g1).

### ChIP-PCR

Wild-type or *Il24*^−/−^ Th17 cells were cultured for 3 d and then fixed at 1 × 10^6^ cells/ml for 10 min using 1% formaldehyde (Thermo Fisher Scientific) at 21°C. Chromatin-protein crosslinking was stopped with 0.125 mol/liter glycine (Merck). Cell lysis and chromatin digestion were performed using SimpleChIP Enzymatic Chromatin IP Kit (Cell Signaling) according to the manufacturer’s instructions. Briefly, 5 × 10^6^ fixed cells per preparation were lysed for 10 min on ice using proprietary detergent buffer containing dithiothreitol and protease inhibitors. Chromatin was digested by incubation with Micrococcal nuclease (1:200; Cell Signaling) for 15 min at 37°C, and nuclear membrane was further dissolved by brief sonication over three cycles (30 s on, 30 s off) in a 4°C cooled Bioruptor Pico (Diagenode). Quality of digested chromatin fragmentation to ∼150 bp in size was determined using a 2100 Bioanalyzer (Agilent) on a high-sensitivity DNA chip. For ChIP, samples were incubated overnight at 4°C with antibodies raised against Histone H3 (clone D2B12, 2.7 µg/IP; Cell Signaling), Stat3 (clone D3Z2G, 0.5 µg/IP; Cell Signaling), or normal rabbit IgG (1 µg/IP; Cell Signaling), followed by 2 h of protein G incubation at 4°C and ascending salinity washes according to the manufacturer’s protocol. Finally, chromatin was eluted at 65°C for 30 min, followed by de-crosslinking of eluted samples and input controls with proteinase K (1:75; Cell Signaling) at 65°C overnight. De-crosslinked chromatin was purified using AMPure XP beads (Beckman Coulter) according to the manufacturer’s instructions. Purified, de-crosslinked chromatin was then subjected to qPCR analysis using SYBR Green (Thermo Fisher Scientific) with proprietary primers for murine RPL30 (Cell Signaling) as a positive control for histone H3 pulling, and published primers for *Il10* (forward 5′-AAC​CCT​AGT​TCC​CAG​AAG​CCA-3′, reverse 3′-CAG​GTG​TCT​CTG​CCT​AGC​CC-5′; Wei et al., 2010) and a gene desert region as negative control (forward 5′-CAA​TGC​ATG​GGT​CCA​GAT​TT-3′, reverse 3′-ATT​GGC​ACG​GAA​GTA​GTG​CT-5′; Giaimo et al., 2017) for Stat3 pulling.

### Microarray

Total RNA was isolated from five independent samples of in vitro–differentiated Th0, Th17, and Th1 cells (d3) using RNeasy Mini Kit (Qiagen). RNA quality was tested on a Bioanalyzer. Using the Ambion WT expression kit, two cycles of cDNA synthesis were performed. Single-stranded cDNA was fragmented and labeled with biotin allonamide triphosphate using the GeneChip WT terminal labeling kit. 5 μg of fragmented and labeled cDNA was hybridized to the GeneChip Mouse Gene 1.0 ST Array. For detailed sample description, see our previously published manuscript (Heinemann et al., 2014). Data analysis was performed using GenePattern open source software (http://genepattern.broadinstitute.org).

### Bulk RNA-seq

Total RNA was isolated from wild-type or *Il24*^−/−^ Th17 cells using the RNAeasy Plus micro kit (74034; Qiagen). Quality and integrity of total RNA was controlled on a Bioanalyzer 2100 (Agilent Technologies). Library preparation for bulk-sequencing of poly(A)-RNA was done as described previously (Parekh et al., 2016). Briefly, barcoded cDNA of each sample was generated with a Maxima RT polymerase (EP0742; Thermo Fisher Scientific) using oligo-dT primer–containing barcodes, unique molecular identifiers (UMIs), and an adaptor. Ends of the cDNAs were extended by a template switch oligo (TSO), and full-length cDNA was amplified with primers binding to the TSO site and the adaptor. NEB UltraII FS kit was used to fragment cDNA. After end repair and A-tailing, a TruSeq adapter was ligated, and 3′-end fragments were amplified using primers with Illumina P5 and P7 overhangs. In comparison to previous descriptions (Parekh et al., 2016), the P5 and P7 sites were exchanged to allow sequencing of the cDNA in read1 and barcodes and UMIs in read2 to achieve better cluster recognition. The library was sequenced on a NextSeq 500 (Illumina) with 67 cycles for the cDNA in read1 and 16 cycles for the barcodes and UMIs in read2. Data were processed using the published Drop-seq pipeline (v1.0) to generate sample- and gene-wise UMI tables (Macosko et al., 2015). Reference genome (GRCm38) was used for alignment. Transcript and gene definitions were used according to GENCODE vM25. Differential gene expression was calculated in R (v4.1.1) using the DESeq2 package (v1.32.0; Love et al., 2014). We focused on genes with <5% probability to be false positive (adjusted P value <0.05).

### Gene set enrichment analysis (GSEA)

GSEA was performed in R (v4.1.1) using the fgsea package (v1.1.18; Korotkevich et al., 2021 *Preprint*). STAT1- and STAT3-regulated gene sets were extracted from a published RNA-seq data set of IL-6–treated wild-type, *Stat1*^−/−^, and *Stat3*^−/−^ CD4^+^ T cells (Hirahara et al., 2015). Genes (among IL-6–regulated genes) positively or negatively regulated by STAT1 or STAT3 were defined as those genes whose log_2_ fold-change (IL-6–treated vs. untreated) in *Stat1*^−/−^ or *Stat3*^−/−^ T cells was smaller than or equal to −0.5 (positive regulation, i.e., down [DN] in knockout) or ≥0.5 (negative regulation, i.e., up [UP] in knockout) compared with the log_2_ fold-change in wild-type cells.

### *Il24* RNA stability assay

The murine thymoma cell line EL4 was cultured in complete DMEM (Hiltensperger et al., 2021). EL4 cells were costimulated under Th17-skewing conditions for 72 h. Cells were then exposed to PBS or IL-1β (25 ng/ml; Miltenyi) in the presence of actinomycin D (2 µg/ml; Tocris Bioscience) for another 16 h of culture.

### Luciferase assays

For *Il24* promoter assays, unstimulated EL4 cells were transfected using a 4D-Nucleofector unit (kit V4XC-2024, protocol CM-120; Lonza) according to the manufacturer’s instructions. For each nucleofection, 2 µg of pXPG plasmid (Addgene) with or without the *Il24* minimal promoter was cotransfected into 1 × 10^6^ EL4 cells alongside 200 ng of Renilla Luciferase Control Reporter Vector pRL-TK (Promega) for normalization. Cells were then costimulated for 48 h with various cytokines to induce specific Th subsets, followed by 4 h of stimulation with PMA (50 ng/ml) and ionomycin (1,000 ng/ml). Cells were lysed and subjected to luciferase analysis using the Dual Luciferase Reporter Assay System (Promega) according to the manufacturer’s instructions. For addressing the regulation of *Il24* mRNA stability through the 3′ UTR, 1 × 10^6^ unstimulated EL4 cells were nucleofected with 2 µg of either pMIR-GLO empty vector (Promega, containing both luciferase and renilla) or vector containing the *Il24* UTR with or without AU-rich regions. Cells were then costimulated for 48 h in the presence of Th-skewing cytokines, lysed, and analyzed using the Dual Luciferase Reporter Assay. All luciferase samples were acquired on a Mithras LB 940 plate reader (Berthold Technologies).

### Quantification and statistical analysis

Statistical evaluations of cell frequency measurements, cell numbers, mRNA amounts, and protein levels were performed with the unpaired Student’s *t* test when two populations were compared. Two-tailed P values <0.05 were considered significant. Multiple comparisons were performed with one-way ANOVA followed by post hoc multiple comparisons tests as indicated in the legends to the figures. EAE curves between groups were analyzed using origin-constrained linear regression as indicated, with difference in slopes tested using two-tailed unpaired *t* test. Calculations and the generation of graphs were performed using Prism v9 (GraphPad software).

### Online supplemental material

Fig. S1 shows that IL-24 is a late cytokine and has no effect on the proliferation of Th17 cells. Fig. S2 shows that IL-24 acts on T cells in a cell-autonomous manner. Fig. S3 shows that neutralization of IL-24 by monoclonal antibody fails to reduce IL-10 expression in T cells in vivo.

## Supplementary Material

SourceData F6 is the source file for Fig. 6.

## Data Availability

Microarray data has been published before (Heinemann et al., 2014) and is available in the Gene Expression Omnibus database under accession no. GSE56021. Th17 STAT3 ChIP-Seq from Durant et al. (2010) was accessed at the Gene Expression Omnibus database under accession no. GSE21669. Data from bulk RNA-seq of *Il24*^+/+^ vs. *Il24*^−/−^ Th17 cells has been made available on the European Nucleotide Archive with accession no. PRJEB48619.
