# Supplementary figures and images for: IL-24 intrinsically regulates Th17 cell pathogenicity in mice
Source: J Exp Med. 2022 Jul 12;219(8):e20212443. doi: 10.1084/jem.20212443 (PMC9280194; doi:10.1084/jem.20212443)

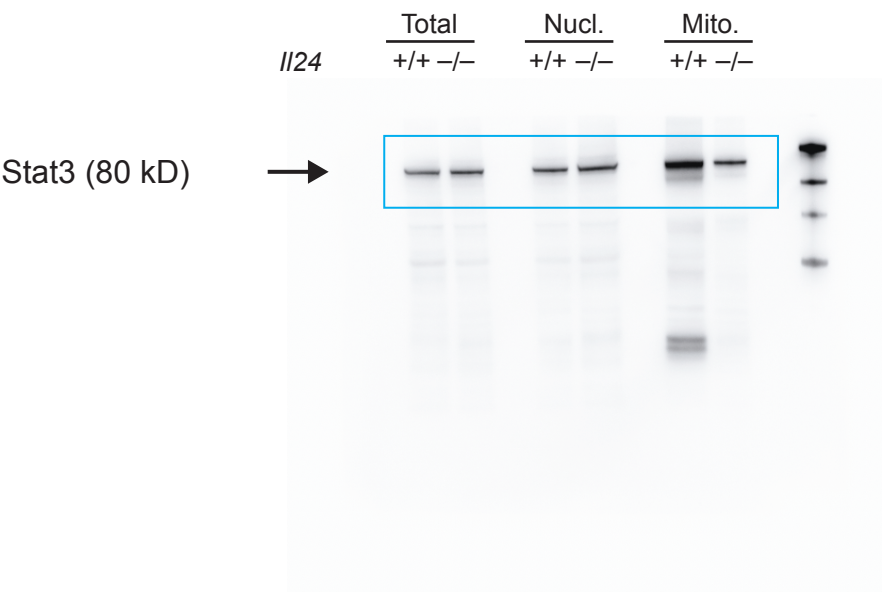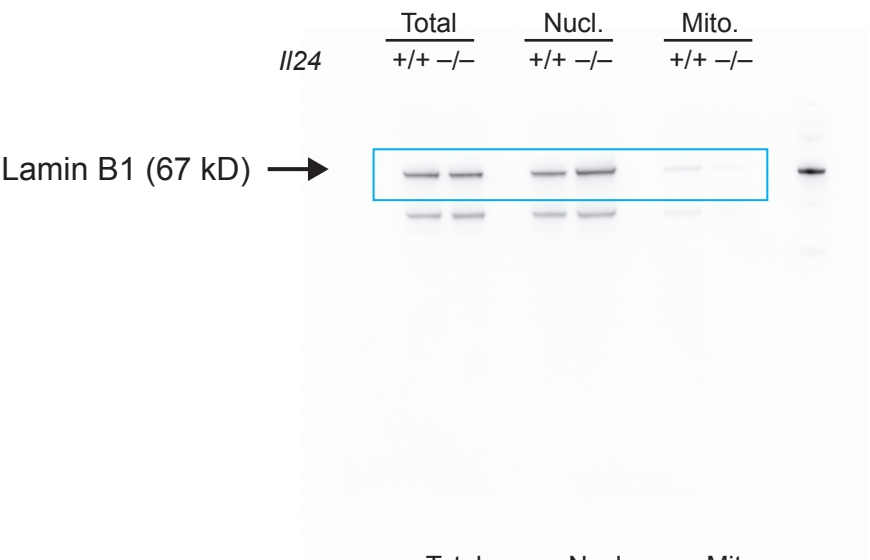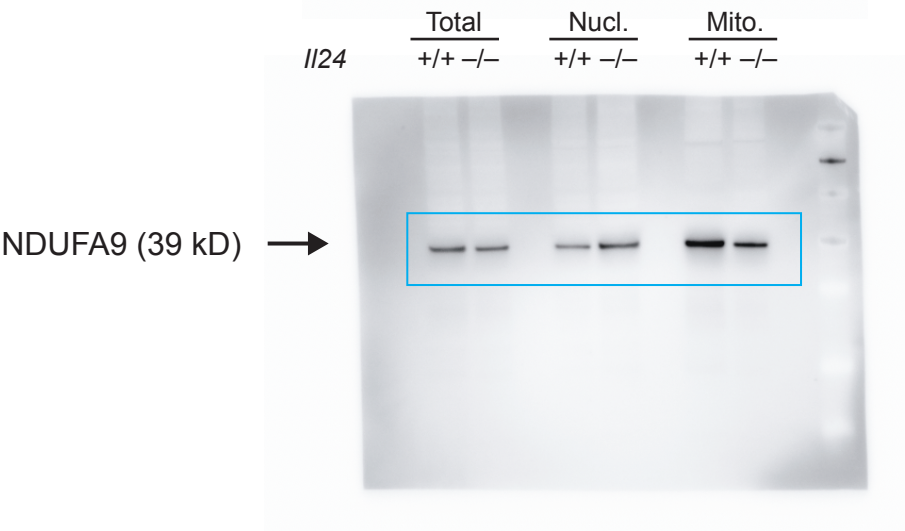

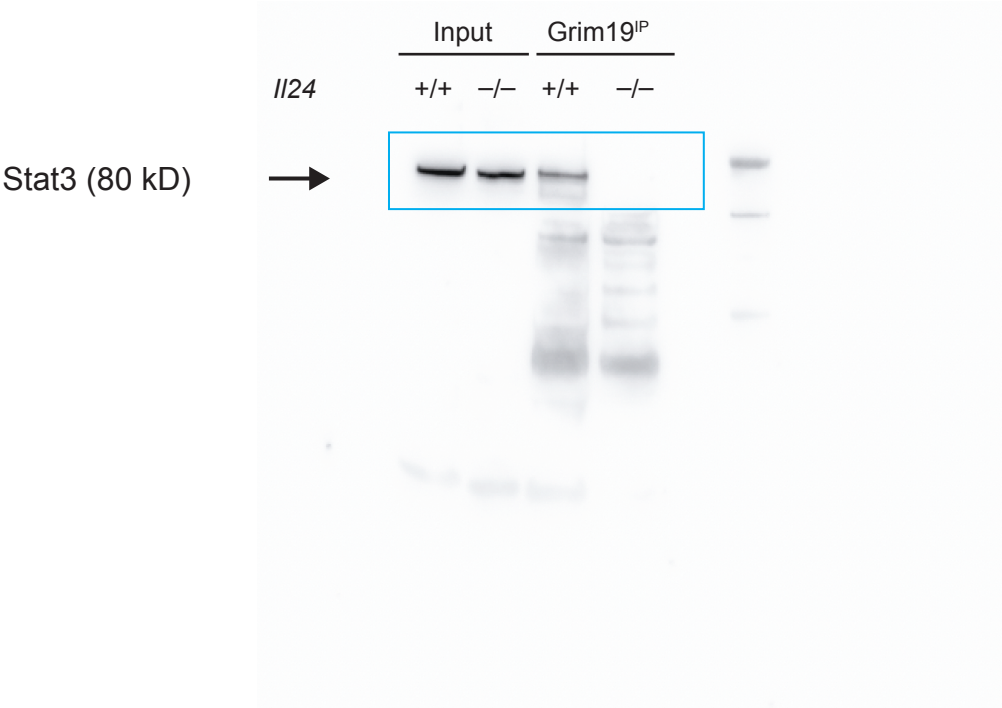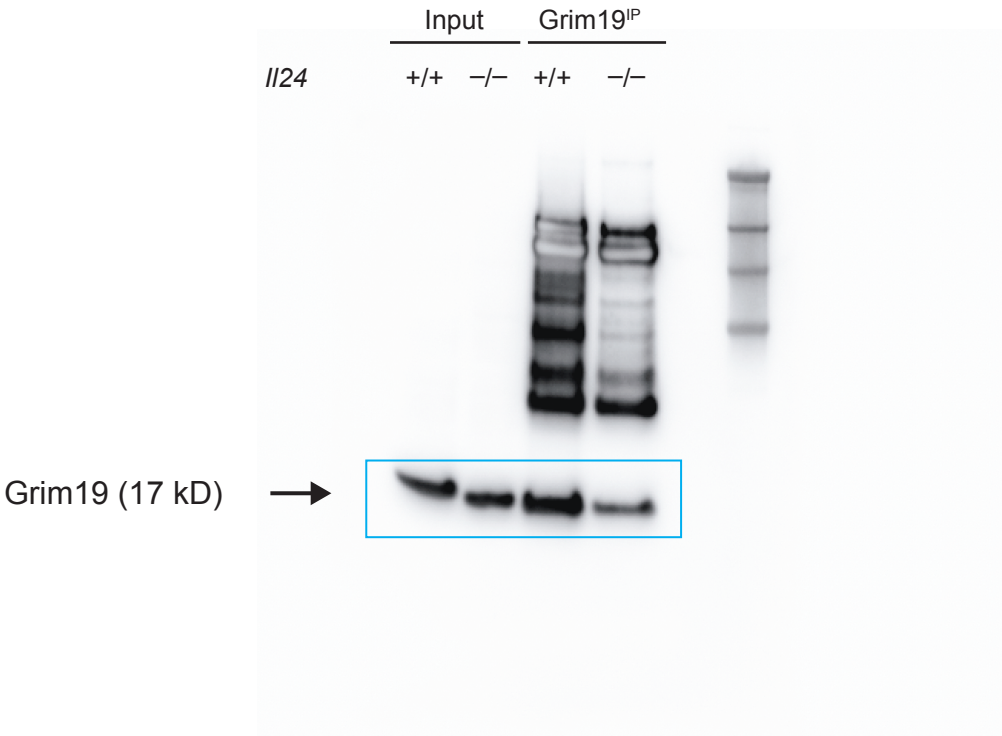

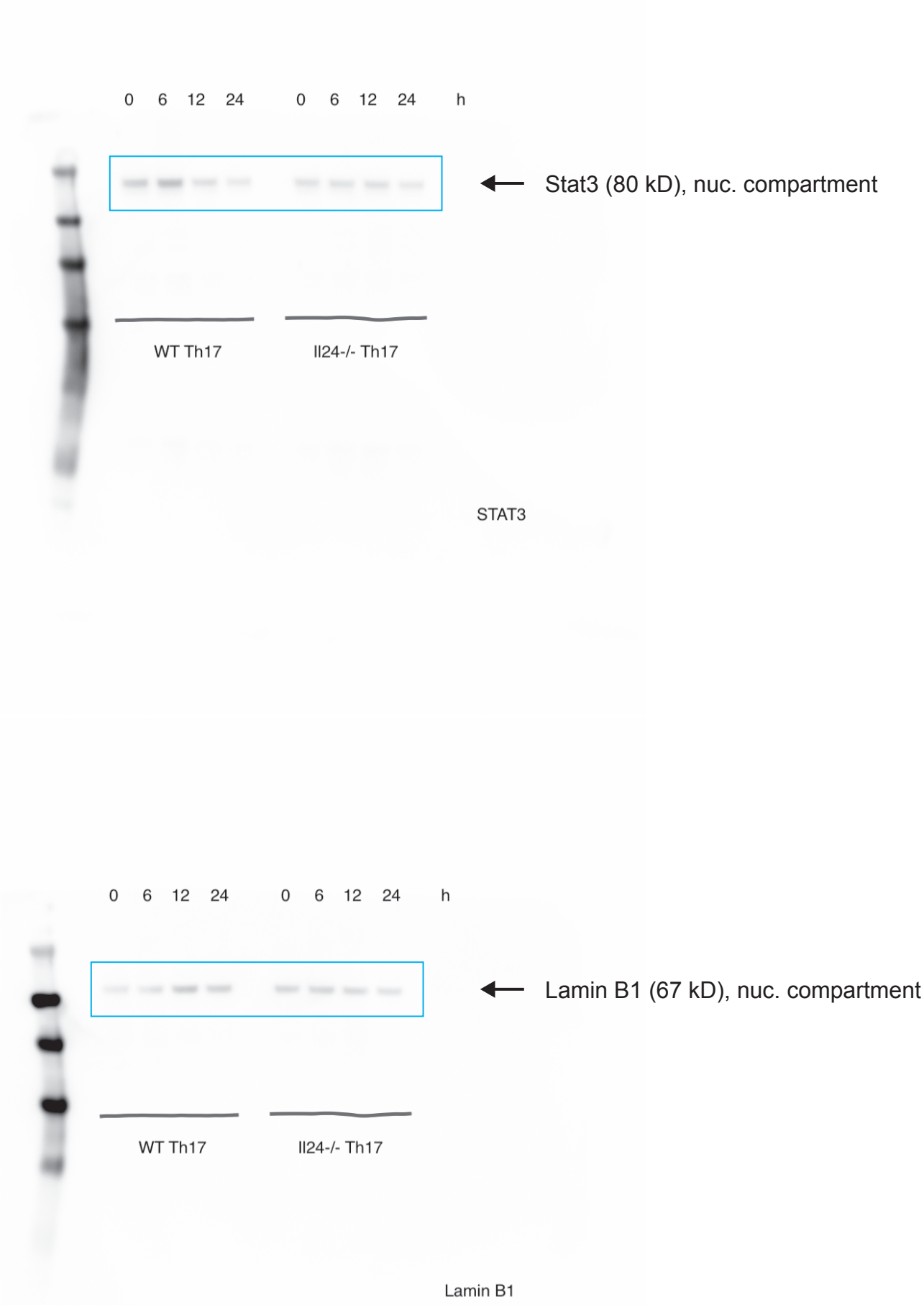

Supplement: SourceData F6 — is the source file for Fig. 6. [file JEM_20212443_SourceDataF6.pdf]
